# Identification of Subtype-Selective Binding Sites in the Opioid Receptor Family

**DOI:** 10.1021/acs.jcim.5c02403

**Published:** 2026-04-03

**Authors:** Antoniel A. S. Gomes, Benoît Guillot, Christian Jelsch, Jesús Giraldo

**Affiliations:** † Laboratory of Molecular Neuropharmacology and Bioinformatics, Unitat de Bioestadística and Institut de Neurociències, 16719 Universitat Autònoma de Barcelona, Bellaterra 08193, Spain; ‡ Unitat de Neurociència Traslacional, Parc Taulí Hospital Universitari, Institut d’Investigació i Innovació Parc Taulí (I3PT), Institut de Neurociències, Universitat Autònoma de Barcelona, Bellaterra 08193, Spain; § Instituto de Salud Carlos III, Centro de Investigación Biomédica en Red de Salud Mental, (CIBERSAM), Madrid 28029, Spain; ∥ CRM^2^, CNRS UMR 7036, Faculté des Sciences et Technologies, 129661 Université de Lorraine, Nancy 54000, France

## Abstract

Selectivity is essential in drug discovery for finding or developing more effective opioid modulators. Structure-based approaches and binding kinetics effectively identify potential druggable regions and determine ligand candidates for improved therapies. In this study, we combined molecular dynamics simulations and funnel-metadynamics with charge density analyses to identify unique structural aspects of the main opioid receptors (ORs), including the mu (μOR), delta (δOR), and kappa (κOR). We found distinct conformational dynamics between the receptors, with the κOR extracellular vestibule tending to form a lid that covers its orthosteric site. Furthermore, we investigated how morphinan-scaffold ligands with distinct pharmacological effects bind to OR orthosteric sites and extracellular vestibules, identifying intermediate ligand states. The lowest-energy states of each complex reproduced the morphinan-like orientation revealed by experimental structures and experimental free energy of binding. Moreover, we determined the role of orthosteric subpockets and extracellular loops (ECLs) in stabilizing ligand-bound states, shedding light on ligand selectivity. Our results provide a detailed description, from an energetic perspective, of the conformational dynamics and structure-based selectivity determination within the OR family. These regions can be rationally targeted for designing and developing functionally selective opioid modulators with improved pharmacological effects in pain treatment or other OR-related diseases.

## Graphical abstract

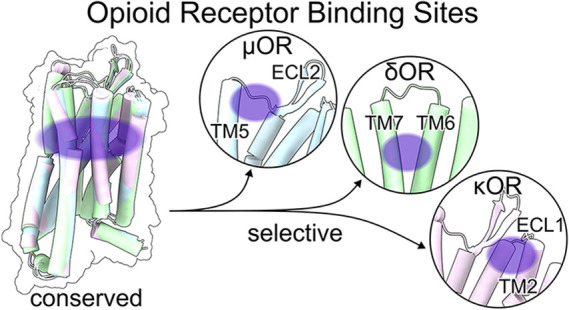

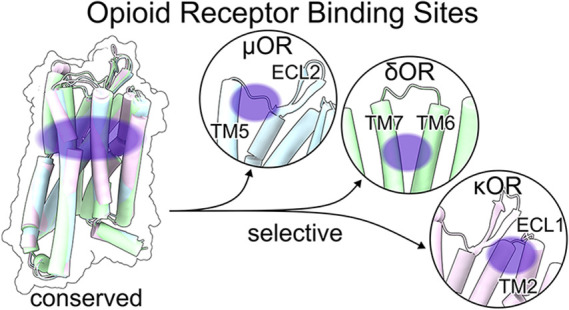

## Introduction

The opioid receptor (OR) family is part of the class A G Protein-Coupled Receptors (GPCRs), comprising the mu (μOR), delta (δOR), kappa (κOR), and nociception (NOP) receptors.^1^ Activation of these receptors by agonists triggers Gi/o protein signaling, which inhibits cyclic AMP (cAMP) production and modulates ion channels, causing neuronal hyperpolarization and reducing neurotransmitter release, resulting in analgesia.^2^ Pain is a condition that includes acute, chronic, neuropathic, and inflammatory pain, among other effects, with all ORs contributing to its relief but differing in their analgesic profiles.^3^ ORs also differ in the magnitude of the side effects they cause, such as respiratory depression, abuse liability, tolerance, constipation, withdrawal symptoms, increased reward, addiction, and dependence, with the μOR displaying the most unwanted effects.^4^ The considerable risk of opioid-induced addiction raises serious medical and socioeconomic concerns.^5^ Data from the Centers for Disease Control and Prevention (CDC) for the 12 months ending in October 2025 revealed over 68,000 overdose deaths in the US, with 64.36% attributed to opioid overdose, primarily to μOR agonists.^6^ These data indicate a progressive decline in opioid overdose deaths since 2023, following the alarming “opioid crisis”, which continues to cause economic consequences.^7^ Although considerably fewer deaths are observed in Europe, similar issues of opioid misuse and overdose are reported,^8^ accounting for approximately 75% of overdose deaths in 2022.^9^ Thus, primarily targeting the μOR for pain relief often leads to several unwanted side effects, which limit the development of safer therapies for pain management but, at the same time, makes evident the need to open new research approaches.^10^

New therapeutics aiming to reduce side effects involve targeting ORs in the peripheral rather than in the central nervous system, tissue-specific pathological conditions, or developing functionally selective opioid agonists.^11,12^ The latter aims to bias OR signaling toward G protein over β-arrestin profiles,^13,14^ although new findings propose a reclassification of these ligands as low-efficacy agonists.^15^ Despite ongoing progress, current evidence suggests that biased agonists show promising results for targeting the δOR or κOR, while low-efficacy ligands targeting the μOR could provide safer pharmacological profiles.^16–19^ Selectively targeting ORs offers opportunities to explore unique signaling pathways while avoiding the secondary effects associated with μOR activation. For instance, δOR modulation promotes antihyperalgesic or antinociceptive effects without tolerance, gastrointestinal erosion, seizures, or respiratory depression.^20,21^ δOR targeting also induces anxiolytic and antidepressant effects with minimal convulsive side effects or abuse liability,^22^ and promising applications for neuropathic pain,^23^ headache,^24^ and brain disorders.^25^ Similarly, selective κOR targeting can provide antinociception without respiratory depression,^26^ analgesia with reduced dysphoria or euphoria,^27^ and low tolerance.^28,29^ κOR modulation has the potential to treat diseases, such as pruritus, multiple sclerosis, Alzheimer’s disease, Parkinson’s disease, immune-mediated diseases, osteoarthritis, and cardiac disorders.^30^

The significant medical interest mentioned above has intensified the search for selective OR modulators. Various classes of μOR selective ligand derivatives have been developed from buprenorphine,^31^ naltrexone,^32^ tramadol,^33^ or fentanyl.^34–36^ Structural studies have provided a comprehensive description of the μOR orthosteric site and subjacent subpockets when bound to several ligands or their derivatives, including DAMGO,^37–39^ fentanyl,^34^ fentanyl-based bitopic ligands,^35,36^ PZM21,^40^ BU72,^41^ FH210,^40^ and mitragynine pseudoindoxyl.^38^ δOR selective ligands have been developed based on morphinan-scaffold,^42–46^ piperazine,^47–49^ piperidine,^50^ spirocyclic,^51^ and quinolinopropellane derivatives.^52^ Ligand derivatives from phenylpiperidine,^53^ morphinan-scaffold,^54^ tetrahydroisoquinolines,^55^ and nalfurafine^56^ have shown selectivity at the κOR. In addition, similar binding poses for JDTic (phenylpiperidine derivative), nalfurafine, and morphinan derivatives revealed a hydrophobic subpocket related to κOR selectivity.^54,57–60^ These studies have extended our structural and functional understanding of OR selectivity, paving the way for personalized opioid drug development using structure-based approaches. In this regard, it is worth highlighting that selectivity should be taken in a broad sense, since opioid analgesics that bind simultaneously to multiple opioid receptors may have improved therapeutic effects and reduced side effects.^3^ This circumstance, which is based on the concept of GPCR polypharmacology,^61^ can be considered to exploit the functional complexity of ORs and become an alternative or complement to single-targeted agents in more ambitious and possibly more realistic multitarget drug discovery programs. Such research strategies require a deep understanding of the commonalities and differences between the binding sites responsible for OR activity. Unraveling the distinctive structure–activity features that characterize these receptors will facilitate the design of novel opioid molecules selectively targeting one or more ORs.

Incorporating binding kinetics and signal activation rates with structure-based approaches can accelerate the rational design of ligands targeting GPCRs.^62^ Structural bioinformatics can explore these aspects through classical or enhanced-sampling Molecular Dynamics (MD) simulation techniques.^63–67^ Among them, metadynamics has been widely used for successfully predicting protein–ligand kinetics, such as residence time, unbinding kinetics of distinct μOR ligands,^64^ GPCR conformational dynamics^68^ and ligand free energy of binding,^69,70^ shedding light on ligand selectivity and intermediate states.^71,72^ In this study, we combine classical MD simulations and metadynamics to explore the dynamical aspects of the three main representatives of the OR family—μOR, δOR, and κOR—and further determine structural aspects related to receptor-subtype selectivity using morphine, buprenorphine, and 17-cyclopropylmethyl-3,14β-dihydroxy-4,5α-epoxy-6α-(isoquinoline-3-carboxamido)­morphinan (NAQ). Their 2D chemical structures are shown in Figure S1. Morphine is an agonist at all three receptors;^73^ NAQ is a partial agonist at the μOR but selective in terms of binding affinity, although it showed relatively high efficacy and moderate potency at the δOR in [^35^S]­GTPγS binding assays;^32,74^ buprenorphine is a partial agonist at the μOR and an antagonist at the δOR and κOR.^75^ This functional variability allowed us to explore the structural determinants of distinct pharmacological profiles within the OR family. We show that these receptors exhibit distinct conformational dynamics, notably through changes in the exposure of their orthosteric sites, which are also influenced by ligands. Additionally, we show how morphinan-scaffold ligands interact with OR electrostatic influence zones, revealing distinct ligand binding modes and receptor regions likely associated with ligand recognition, thus providing a structural basis for rationally designing ligands that selectively target the OR family. An overview of all simulations performed in our work is available in Table S1. OR residues are identified according to Ballesteros–Weinstein numbering as superscript.^76^

## Results

### OR Orthosteric Sites Exhibit Distinct Dynamics

We studied the dynamics of the μOR, δOR, and κOR using microsecond-timescale MD simulations to capture distinct conformational aspects of these receptors. Our results indicate notable conformational changes in the extracellular vestibules of these receptors. These changes were analyzed by calculating the volume of their orthosteric sites and the extracellular vestibules using Epock^77^ and comparing them with their respective experimental structures to obtain the relative volume of each receptor (Figure 1). We found that the μOR and δOR maintained their relative volumes, respectively showing a slight increase and decrease of 10% compared to their experimental structure (Figure 1A). We observed that the κOR tended to form a lid above its orthosteric site, showing a 20% volume reduction compared to its respective experimental structure (Figure 1A), likely due to the existence of a salt bridge between R202^ECL2^ and E^6.58^ residues (Figure 1B). Although the δOR also presented a lid through a salt bridge between D193^ECL2^ and R291^ECL3^ residues (Figure 1C), these interactions did not affect the volume of its orthosteric site. The minimum distances of these pairs of residues during MD simulations are shown in Figure S2.

**Figure 1:**
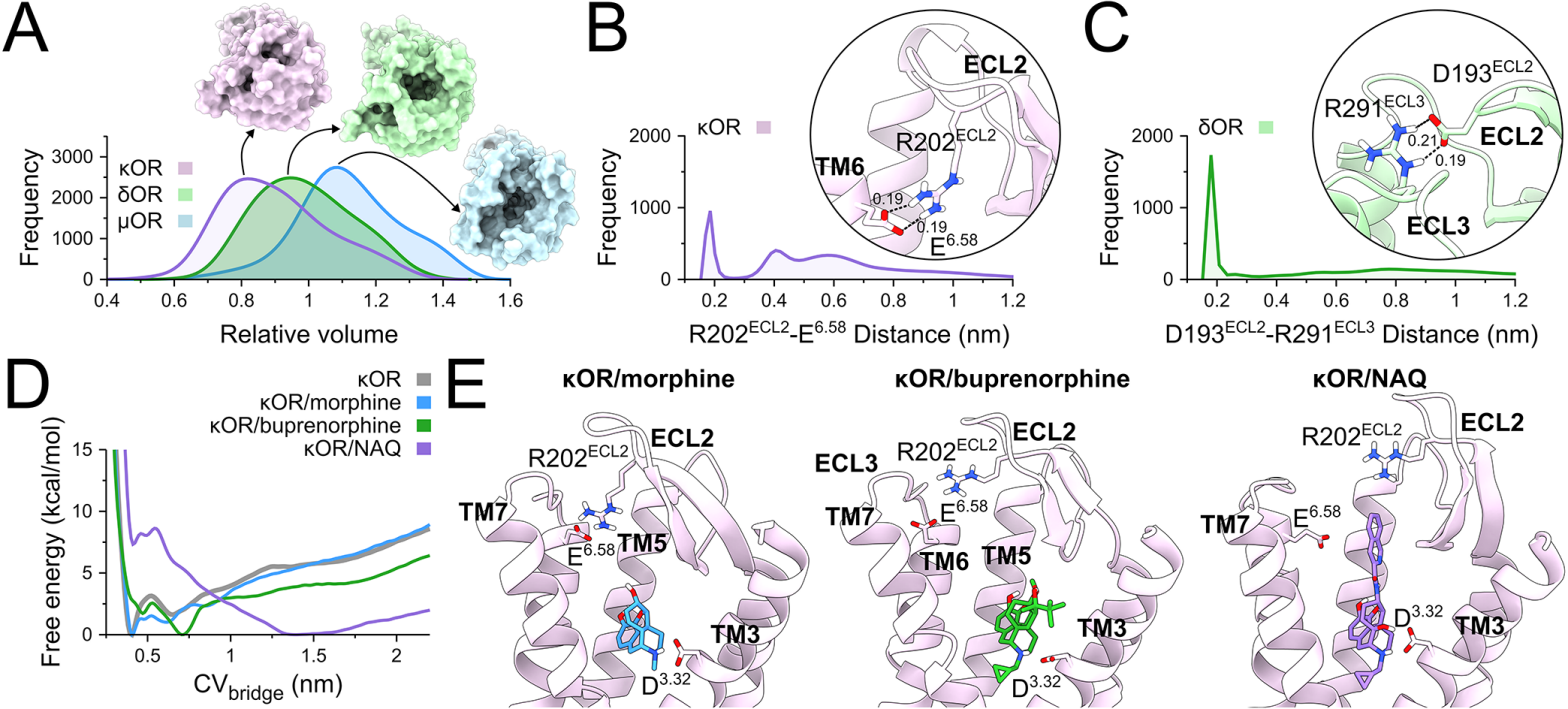
Structural aspects of the OR family. (A) Classical MD simulations assessed the dynamics of OR orthosteric sites by measuring the relative volume of their experimental structures. μOR, δOR, and κOR surfaces display the extracellular view of representative conformations of their most frequent relative volumes. (B) The frequency of the minimum distance between R202^ECL2^ and E^6.58^ in the κOR and (C) D193^ECL2^ and R291^ECL3^ in the δOR is shown, highlighting their respective positions in receptor structures. (D) Free energy landscapes of the κOR salt bridge distance, CV_bridge_, are shown for unbound or bound states to morphine, buprenorphine, or NAQ, revealing (E) representative lowest-energy conformations for each complex. The μOR, δOR, and κOR are respectively colored light blue, light green, and light purple, while morphine, buprenorphine, and NAQ are respectively colored blue, green, and purple. TM or ECL portions were omitted for clarity in visualization.

### Ligands Modulate the κOR Orthosteric Site Dynamics

The reduction in the relative volume of the κOR orthosteric site in our simulations, driven by the salt bridge between R202^ECL2^ and E^6.58^, motivated us to investigate its role in receptor structure and how ligands impact it. Therefore, we defined a collective variable (CV) to explore the distance between the CZ atom of R202^ECL2^ and the CD atom of E^6.58^, termed CV_bridge_ (Figure S3A). This new variable (Figure S3B) followed the receptor frequency distribution from the classical MD simulation results, previously calculated by the minimum distance of these residues (Figure 1B), indicating that CV_bridge_ can appropriately capture these conformational aspects. We then performed metadynamics^78^ to determine the free energy landscape of CV_bridge_ of the κOR unbound and bound to morphine, buprenorphine, and NAQ.

Metadynamics identified two low-energy regions in CV_bridge_, with the lowest-energy state at 0.41 nm represented by a stable salt bridge between R202^ECL2^ and E^6.58^ κOR residues, which agrees with our results obtained from classical MD (Figure 1D, gray line). This conformational state presented R202^ECL2^ embedded in a polar region containing E209^ECL2^ and S303^ECL3^ residues (Figure S3C). The second lowest-energy state, identified at 0.65 nm, did not form a salt bridge between R202^ECL2^ and E^6.58^. Instead, these residues were in contact with a network composed of E209^ECL2^, T302^ECL3^, and S303^ECL3^ residues (Figure S3D).

The presence of ligands bound to the κOR induced changes in the CV_bridge_ free-energy landscape. While morphine induced similar states (Figure 1D, blue line) to those observed in the unbound receptor, buprenorphine induced a shift of the lowest-energy state of CV_bridge_ to 0.70 nm (Figure 1D, green line). The conformations of these complexes are similar to those observed in the unbound receptor, with the same pattern observed in the presence of morphine (Figure S3E). In contrast, the state around 0.70 nm showed D204^ECL2^ stabilizing R202^ECL2^ in the presence of buprenorphine (Figure S3F). NAQ completely abolished these low-energy states in the κOR, preventing interactions between R202^ECL2^ and E^6.58^ residues, shifting the low-energy state to 1.39 nm (Figure 1D, purple line). Interestingly, this region corresponds to a plateau in CV_bridge_, representing the experimental open conformation, with a CV_bridge_ value of 1.67 nm. Thus, NAQ maintains the κOR around the experimental conformation, imposing an energy barrier of 8.95 ± 0.28 kcal/mol on salt bridge formation between R202^ECL2^ and E^6.58^ residues, with a minimum of 8.47 ± 0.15 kcal/mol. In the plateau region between 1.4 and 1.6 nm, similar energy values were observed for κOR unbound and morphine-bound of −6.41 ± 0.07 and −6.47 ± 0.06 kcal/mol, respectively. In contrast, a slightly reduced value of −5.27 ± 0.05 kcal/mol was observed when the receptor was bound to buprenorphine. Figure 1E shows the different orientations of R202^ECL2^ and E^6.58^ residues in the presence of morphine, buprenorphine, and NAQ. The time-dependent free energy values and free energy profiles for each system are shown in Figure S4.

### Using Funnel-Metadynamics to Explore Ligand Binding States in ORs

Funnel-metadynamics is an enhanced-sampling technique that accurately reproduces ligand-free energy of binding and extensively explores ligand interactions around protein binding sites, successfully applied to GPCRs.^69,71,72^ Here, we used this technique to investigate how morphine, buprenorphine, and NAQ bind to ORs. We employed well-tempered funnel-metadynamics^78–80^ for each complex along CVs projected onto the XY-plane (XY-projection) and the *Z*-axis (Z-projection), allowing ligands to explore the entire orthosteric site and extracellular vestibules of the receptors, revealing low-energy and intermediate states. Due to the lid formed in the κOR, we included CV_bridge_ as a third CV for a proper convergence of the free energy (refer to Methods section). We stress that, although ECL2 and ECL3 residues formed a lid in the δOR, these interactions did not prevent ligand exploration nor affect free energy of binding calculations. We verified the data convergence after obtaining an asymptotic free energy curve in the last 200 ns of metadynamics simulations. All calculated free energy of binding values (Δ*G*_calc_) in this work were accurately reproduced within the experimental (Δ*G*_exp_) range (Table 1). The time-dependent free energy values and free energy profiles of each complex are shown in Figures S5 and S6, respectively.

**Table 1:** Experimental and Calculated Free Energies of Binding (Kcal/Mol) of Ligands Bound to ORs^a^

	**Morphine**		**Buprenorphine**		**NAQ**	
	Δ*G*_exp_	Δ*G*_calc_	Δ*G*_exp_	Δ*G*_calc_	Δ*G*_exp_	Δ*G*_calc_
μOR	–12.30 ^81^, –11.23 ^82^	–11.74 ± 0.13	–13.27 ^83^, –12.11 ^81^	–12.58 ± 0.34	–12.71 ^32^, –12.29 ^74^	–12.28 ± 0.09
δOR	–9.41 ^81^, –9.15 ^82^	–9.43 ± 0.16	–11.46 ^81^, –11.28 ^84^	–11.40 ± 0.13	–9.44 ^32^, –9.32 ^74^	–9.39 ± 0.23
κOR	–10.06 ^81^, –9.54 ^82^	–10.06 ± 0.11	–12.49 ^81^, –9.54 ^84^	–12.24 ± 0.24	–10.81 ^74^, –10.40 ^32^	–10.40 ± 0.15

a Experimental inhibitory affinity constants (K_i_) of each complex were converted to free energies from the relation Δ*G* = RT ln­(K_i_) at *T* = 300 K. All computed values were corrected for the standard volume and funnel potentials as described in Methods section.

We then inspected the funnel-metadynamics results and selected three relevant binding states (S1, S2, and S3) from each complex, revealing their interaction modes from an energetic perspective. Further, we selected ensembles of structures corresponding to each state to provide a global description of the most relevant OR residues for stabilizing morphine, buprenorphine, or NAQ.

### Ligand Binding to the μOR

The lowest-energy binding pose, S1, showed a similar binding mode for all ligands in the μOR orthosteric site (Figure 2). Δ*G*_calc_ values were −11.74 ± 0.13, −12.58 ± 0.34, and −12.28 ± 0.09 kcal/mol for morphine, buprenorphine, and NAQ, respectively (Table 1). All ligands engaged in frequent contacts with μOR residues in TM3, such as D^3.32^ and Y^3.33^, including surrounding residues such as M^3.36^, V^5.42^, W^6.48^, I^6.51^, V^6.55^, W^7.35^, I^7.39^, and Y^7.43^ (Figures 2 and S7). This state corresponds to the same orientation observed experimentally for the complex μOR/morphine (PDB ID: 8EF6),^34^ presenting a root mean square deviation (RMSD) of 0.21 ± 0.05 nm for non-hydrogen atoms of morphine after aligning backbone atoms of the receptor. We identified that the buprenorphine’s 2-hydroxy-3,3-dimethylbutan-2-yl and 6-O-methyl ether moieties preferentially interacted with Q^2.60^ and I^3.29^ residues compared to morphine and NAQ (Figures 2 and S7). These chemical groups projected toward the extracellular vestibules of the receptor, stabilizing interactions with L221^ECL2^ (Figures 2 and S7). Interestingly, the NAQ’s isoquinoline group projected farther than buprenorphine, interacting with a hydrophobic region in ECL2 (T220^ECL2^, L221^ECL2^, and F223^ECL2^), and with E^5.35^, K^6.58^, A^6.59^, and T309^ECL3^ residues (Figures 2 and S7).

**Figure 2:**
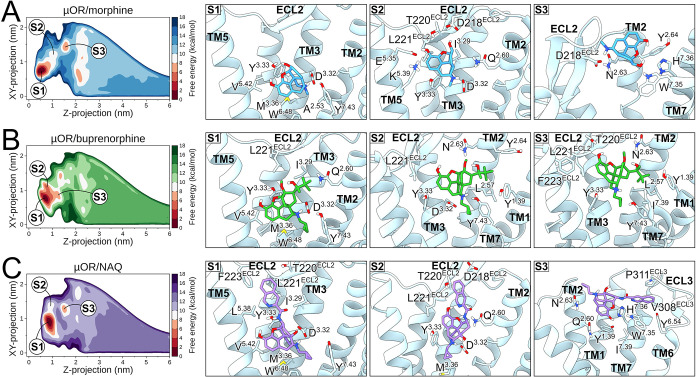
Ligand binding modes of the μOR. The left panels display free-energy landscapes of S1, S2, and S3 states of the μOR bound to (A) morphine, (B) buprenorphine, and (C) NAQ using sequential palettes of blue, green, and purple, respectively. Red sequential palettes color low-energy regions. The right panels show representative structures of ligand states bound to the μOR, highlighting receptor residues in contact with the ligands. The μOR is represented as light blue cartoons or sticks, while morphine, buprenorphine, and NAQ are colored blue, green, and purple sticks. Oxygen, nitrogen, sulfur, and polar hydrogen atoms are colored red, blue, yellow, and white, respectively. TM or ECL portions were omitted for clarity in visualization.

The second energy minimum, S2, was also similar for all ligands, located laterally and slightly above S1, maintaining interactions with D^3.32^ (morphine and NAQ) or Y^7.43^ (buprenorphine) (Figure 2). The S2 state presented Δ*G*_calc_ values of 8.04 ± 0.22, 5.25 ± 0.28, and 9.31 ± 0.15 kcal/mol higher than S1 for morphine, buprenorphine, and NAQ, respectively (Table 1), allowing the ligands to interact with TM3 and ECL2 residues such as I^3.29^, D^3.32^, Y^3.33^, T220^ECL2^, and L221^ECL2^ (Figures 2 and S7). Additionally, TM5 residues accommodated morphine (E^5.35^ and K^5.39^) and NAQ (V^5.42^), while TM6 residues stabilized buprenorphine (W^6.48^ and I^6.51^) and NAQ (W^6.48^, I^6.51^, and K^6.58^) (Figures 2 and S7). Furthermore, the *N*-methylcyclopropyl group, found in buprenorphine and NAQ, interacted with TM7 residues, including I^7.39^, G^7.42^, and Y^7.43^ (Figures 2 and S7). The presence of the buprenorphine’s 2-hydroxy-3,3-dimethylbutan-2-yl group facilitated its projection toward the receptor side, interacting with residues in TM1 (I^1.35^, M^1.36^, and Y^1.39^), TM2 (T^2.56^, L^2.57^, Q^2.60^, S^2.61^, N^2.63^, and Y^2.64^), TM6 (W^6.48^ and I^6.51^), and TM7 (W^7.35^, H^7.36^, I^7.39^, G^7.42^, and Y^7.43^) (Figures 2 and S7).

The third energy minimum, S3, was located above all other states while presenting distinct ligand binding modes (Figure 2). This state presented Δ*G*_calc_ values of 6.01 ± 0.30, 7.36 ± 0.38, and 7.08 ± 0.36 kcal/mol higher than S1 for morphine, buprenorphine, and NAQ, respectively (Table 1). Morphine mainly interacted with TM2 and TM7 residues, including N^2.63^, Y^2.64^, G^2.67^, W^7.35^, and H^7.36^, as well as ECL residues T134^ECL1^, W135^ECL1^, R213^ECL2^, Q214^ECL2^, S216^ECL2^, I217^ECL2^, and D218^ECL2^ (Figures 2 and S7). Buprenorphine interacted with residues in TM1 (Y^1.39^), TM2 (L^2.57^, Q^2.60^, and N^2.63^), TM3 (I^3.29^, D^3.32^, and Y^3.33^), ECL2 (C219^ECL2^, T220^ECL2^, L221^ECL2^, and F223^ECL2^), TM5 (E^5.35^ and K^5.39^), TM6 (I^6.51^ and V^6.55^), and TM7 (W^7.35^, H^7.36^, I^7.39^, and Y^7.43^) (Figures 2 and S7). NAQ interacted with TM1 (Y^1.39^), TM2 (Q^2.60^, N^2.63^, Y^2.64^, and G^2.67^), ECL1 (T134^ECL1^ and W135^ECL1^), ECL2 (R213^ECL2^, S216^ECL2^, I217^ECL2^, D218^ECL2^, and C219^ECL2^), TM6 (Y^6.54^, I^6.57^, and K^6.58^), ECL3 (T309^ECL3^, I310^ECL3^, and P311^ECL3^), and TM7 (Q^7.31^, T^7.32^, S^7.34^, W^7.35^, H^7.36^, and I^7.39^) (Figures 2 and S7).

### Ligand Binding to the δOR

As with μOR, the lowest-energy binding pose, S1, of all three ligands presented similar binding modes at the δOR orthosteric site (Figure 3). Δ*G*_calc_ values of −9.43 ± 0.16, −11.40 ± 0.13, and −9.39 ± 0.23 kcal/mol were found for morphine, buprenorphine, and NAQ (Table 1), respectively. The classical interaction with D^3.32^ and Y^3.33^ was observed with high frequency in all ligands, including neighboring residues in TM3 (M^3.36^), TM5 (K^5.39^ and V^5.42^), TM6 (W^6.48^, I^6.51^, H^6.52^, and V^6.55^), and TM7 (I^7.39^) (Figures 3 and S8). While morphine did not present specific contacts in this state, buprenorphine and NAQ engaged in notable interactions with TM2 (A^2.53^, Q^2.60^, and K^2.63^) and ECL2 residues (Figures 3 and S8). This latter region interacted with buprenorphine and NAQ through C198^ECL2^, M199^ECL2^, and L200^ECL2^, while NAQ specifically interacted with R192^ECL2^ and V197^ECL2^ residues (Figures 3 and S8). The *N*-methylcyclopropyl group of buprenorphine and NAQ interacted with G^7.42^ and Y^7.43^, located deep within the δOR orthosteric site (Figures 3 and S8).

**Figure 3:**
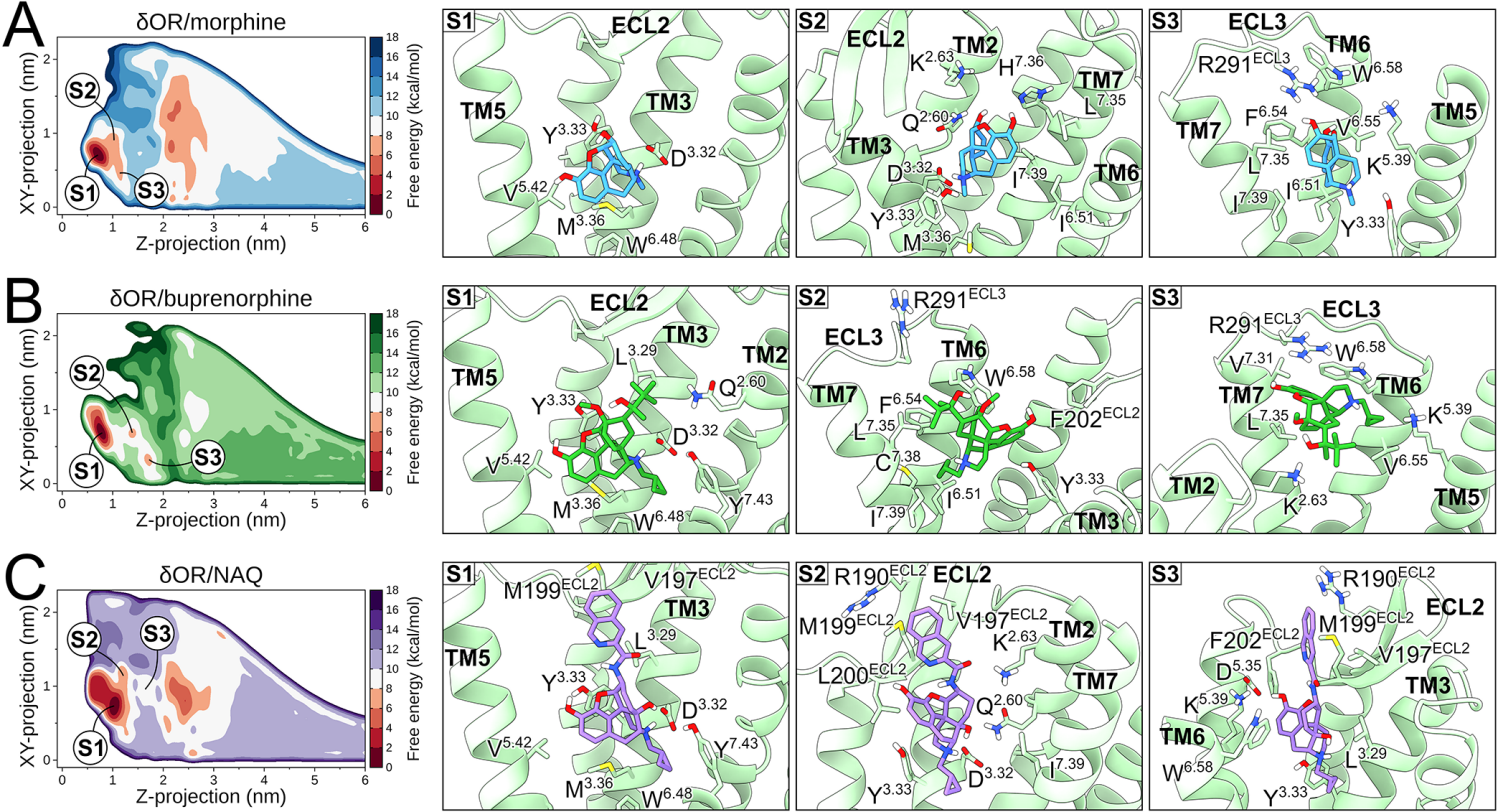
Ligand binding modes of the δOR. The left panels display free-energy landscapes of S1, S2, and S3 states of the δOR bound to (A) morphine, (B) buprenorphine, and (C) NAQ using sequential palettes of blue, green, and purple, respectively. Red sequential palettes color low-energy regions. The right panels show representative structures of ligand states bound to the δOR, highlighting receptor residues in contact with the ligands. The δOR is represented as light green cartoons or sticks, while morphine, buprenorphine, and NAQ are colored blue, green, and purple sticks. Oxygen, nitrogen, sulfur, and polar hydrogen atoms are colored red, blue, yellow, and white, respectively. TM or ECL portions were omitted for clarity in visualization.

The second energy minimum, S2, presented the ligands placed slightly above S1 (Figure 3), with Δ*G*_calc_ values of 6.54 ± 0.79, 7.86 ± 0.61, and 5.70 ± 0.30 kcal/mol higher than S1, respectively, for morphine, buprenorphine, and NAQ (Table 1). Although all ligands interacted with Y^3.33^, only morphine and NAQ maintained contact with D^3.32^ (Figures 3 and S8). This missing interaction was due to a rearrangement of buprenorphine inside the δOR orthosteric site, where its 2-hydroxy-3,3-dimethylbutan-2-yl and *N*-methylcyclopropyl groups interacted with a subpocket formed by TM6 and TM7 residues, including I^6.51^, F^6.54^, V^6.55^, W^6.58^, L^7.35^, and I^7.39^ (Figures 3 and S8). Morphine occupied the same subpocket despite reduced interactions with I^6.51^, L^7.35^, and I^7.39^ compared to buprenorphine. Conversely, NAQ occupied a region near TM2, interacting with Q^2.60^, K^2.63^, and L^3.29^, including ECL2 residues, such as E190^ECL2^, R192^ECL2^, V197^ECL2^, C198^ECL2^, M199^ECL2^, L200^ECL2^, and F202^ECL2^. Buprenorphine also interacted with some of these regions, notably with ECL2 residues (Figures 3 and S8).

### Ligand Binding to the κOR

In the third opioid receptor studied in this work, we observed similar binding modes at the κOR orthosteric site for the lowest-energy binding pose, S1 (Figure 4). Δ*G*_calc_ values of −10.06 ± 0.11, −12.24 ± 0.24, and −10.40 ± 0.15 kcal/mol were found for morphine, buprenorphine, and NAQ, respectively (Table 1). Again, all ligands interacted with D^3.32^ and Y^3.33^, along with residues in TM2 (V^2.53^ and Q^2.60^), TM3 (M^3.46^), TM5 (K^5.39^ and V^5.42^), TM6 (W^6.48^, I^6.51^, H^6.52^, and I^6.55^), and TM7 (Y^7.35^, I^7.39^, G^7.42^, and Y^7.43^) (Figures 4 and S9). Buprenorphine and NAQ also presented specific interactions with κOR residues in ECL2, including C210^ECL2^, S221^ECL2^, and L212^ECL2^. A specific hydrophobic subpocket in the κOR accommodated the buprenorphine’s 2-hydroxy-3,3-dimethylbutan-2-yl group, facilitating interactions with F^2.59^, V^2.63^, W124^ECL1^, V^3.28^, and I^3.29^ (Figures 4 and S9). As with μOR, the NAQ’s isoquinoline group projected toward the κOR extracellular vestibule, resulting in specific interactions with ECL2 and TM5 residues, such as R202^ECL2^, E209^ECL2^, F214^ECL2^, Y^5.31^, and D^5.35^ (Figures 4 and S9).

**Figure 4:**
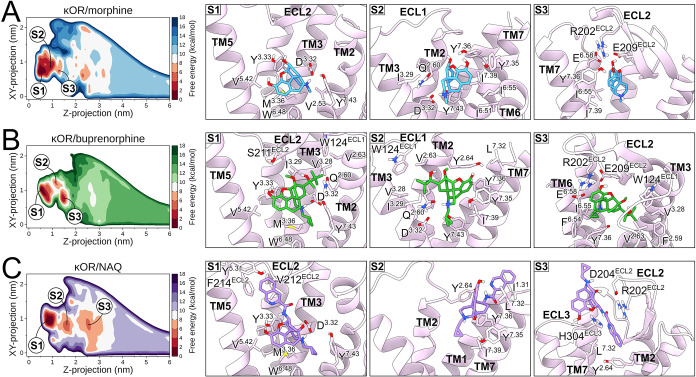
Ligand binding modes of the κOR. The left panels display free-energy landscapes of S1, S2, and S3 states of the κOR bound to (A) morphine, (B) buprenorphine, and (C) NAQ using sequential palettes of blue, green, and purple, respectively. Red sequential palettes color low-energy regions. The right panels show representative structures of ligand states bound to the κOR, highlighting receptor residues in contact with the ligands. The κOR is represented as light purple cartoons or sticks, while morphine, buprenorphine, and NAQ are colored blue, green, and purple sticks. Oxygen, nitrogen, sulfur, and polar hydrogen atoms are colored red, blue, yellow, and white, respectively. TM or ECL portions were omitted for clarity in visualization.

The second energy minimum, S2, presented Δ*G*_calc_ values of 2.20 ± 0.18, 5.13 ± 0.48, and 3.93 ± 0.22 kcal/mol higher than S1 for morphine, buprenorphine, and NAQ, respectively (Table 1). The free energy surface indicates that morphine explores a wide range of values around the lowest-energy minimum, allowing the identification of a second (S2) low-energy state energetically similar to S1 (Figures 4 and S9). This state indicates that morphine preserves several interactions within the κOR orthosteric site, including polar contacts with D^3.32^ and hydrophobic interactions with I^6.51^ and I^6.55^, also projecting toward TM7 to form frequent polar interactions with Y^7.35^ and Y^7.36^ (Figures 4 and S9). Although buprenorphine also presents a similar orientation, its interactions with TM6 were almost abolished in the S2 state compared to morphine. We observed hydrophobic interactions involving buprenorphine and TM7 residues Y^7.35^, Y^7.36^, I^7.39^, and Y^7.43^, including those in the subpocket identified in S1 (Figures 4 and S9). NAQ was located higher than the other ligands in the S2 state, interacting with residues in TM1 (I^1.31^ and P^1.32^), TM2 (V^2.63^ and Y^2.64^), and TM7 (T^7.29^, L^7.32^, S^7.33^, Y^7.35^, Y^7.36^, I^7.39^, and Y^7.43^) (Figures 4 and S9).

In the third energy minimum, S3, Δ*G*_calc_ values of 4.97 ± 0.13, 1.32 ± 0.56, and 5.03 ± 0.21 kcal/mol higher than S1 were found for morphine, buprenorphine, and NAQ, respectively (Table 1). In this state, despite the absence of interactions with D^3.32^, morphine participated in polar interactions with Y^7.35^ and Y^7.36^ as found in S2, including the additional participation of the charged residues R202^ECL2^, E209^ECL2^, and E^6.58^ (Figures 4 and S9). In the case of buprenorphine, its S3 state remained interacting with the hydrophobic subpocket identified in S1 and S2 states, involving TM2, ECL1, and TM3 residues (Figures 4 and S9). Additional hydrophobic interactions involving TM6 (F^6.54^ and I^6.55^) and TM7 (L^7.32^ and Y^7.35^) residues, along with polar interactions with ECL2 charged residues, stabilized buprenorphine in the S3 state (Figure 4 and S9). The intrinsic dynamics to form a lid over the κOR orthosteric site facilitated the availability of these charged residues for stabilizing morphine and buprenorphine, as shown in Figures 1 and S3. In S3, NAQ was oriented upside down at the entrance of the κOR orthosteric site, interacting with TM1 (P^1.32^), TM2 (Y^2.64^ and L^2.65^), ECL2 (R202^ECL2^, D204^ECL2^, V205^ECL2^, and E209^ECL2^), ECL3 (H304^ECL3^), and TM7 (S^7.28^, T^7.29^, and L^7.32^) residues (Figures 4 and S9).

### Toward a Comprehensive Description of Ligand Selectivity at ORs

The extended ligand spatial exploration performed by funnel-metadynamics allowed a detailed investigation of OR orthosteric sites and extracellular vestibules. We concatenated these data to unveil structural aspects at the μOR, δOR, and κOR likely involved in ligand recognition, providing structural insights into ligand selectivity within the OR family. Thus, we highlighted the residues of each experimental OR structure according to their percentages of contacts in the binding of morphine, buprenorphine, and NAQ (Figure S10). Globally, we identified TM3, TM5, TM6, and TM7 residues as essential in all receptors, indicating the importance of their orthosteric sites in ligand stabilization, followed to a lesser extent by TM2 and TM1 (Figure S10). We found that ECL2 is essential for recognizing ligands in all receptors, notably through the β-sheet region above their orthosteric sites, while ECL1 and ECL3 residues participate less in ligand binding.

Upon detailed analysis, we identified μOR binding sites located in TM2, ECL1, and ECL2 that stabilize morphine (Figure S10A), buprenorphine (Figure S10B), and NAQ (Figure S10C). Conversely, we identified δOR binding regions located in TM5, TM6, and TM7, for morphine (Figure S10D), buprenorphine (Figure S10E), and NAQ (Figure S10F). In the κOR, we found that TM6 and TM7 residues were of high importance in ligand recognition, including the ECL2 region closest to the orthosteric site for morphine (Figure S10G), buprenorphine (Figure S10H), and NAQ (Figure S10I). These results also highlight preferential ligand regions in ORs, where buprenorphine interacts with a hydrophobic subpocket in the κOR formed by TM2 and ECL1 residues (Figure S10H). Furthermore, significant interactions of NAQ were observed with an ECL2 region near TM5 in the μOR (Figure S10C), δOR (Figure S10F), and κOR (Figure S10I).

These results motivated us to examine the structural features of each OR. We then superposed OR experimental structures and identified three regions with distinct residue compositions, which correspond to subtype-selective binding sites, namely a, b, and c (Figure 5). Region a is located between ECL2 and TM5, where we identified a hydrophobic subpocket in all receptors, composed of leucine and phenylalanine residues (L221^ECL2^ and F223^ECL2^ in the μOR, L200^ECL2^ and F202^ECL2^ in the δOR, and L212^ECL2^ and F214^ECL2^ in the κOR). Near these residues, a negatively charged residue is found at position 5.35 in all receptors, with a long side chain (E^5.35^) in the μOR and a short side chain (D^5.35^) in the δOR and κOR (Figure 5, region a). In addition, position 5.31 features an aromatic residue in the κOR instead of a polar, short side chain residue in the μOR and δOR (Figure 5, region a). We performed an additional set of funnel-metadynamics simulations to test whether these positions affect the affinity to the μOR. Thus, we generated mutated versions of the μOR at positions 5.31 and 5.35 to match those present at δOR and κOR. These mutants were named μOR-SD and μOR-YD (see Methods section). The time-dependent free energy values and free energy profiles of NAQ binding to these new μOR mutants are shown in Figure S11, and the free-energy landscapes are shown in Figure S12. Interestingly, our funnel-metadynamics results showed lower free energy of binding compared to the wild-type receptor, with values of −9.83 ± 0.17 and −10.10 ± 0.09 kcal/mol for μOR-SD and μOR-YD, respectively. Of note, these values are close to those found for the binding of NAQ to wild-type δOR and κOR (Table 1). This provides direct computational support for the proposed selectivity mechanism.

**Figure 5:**
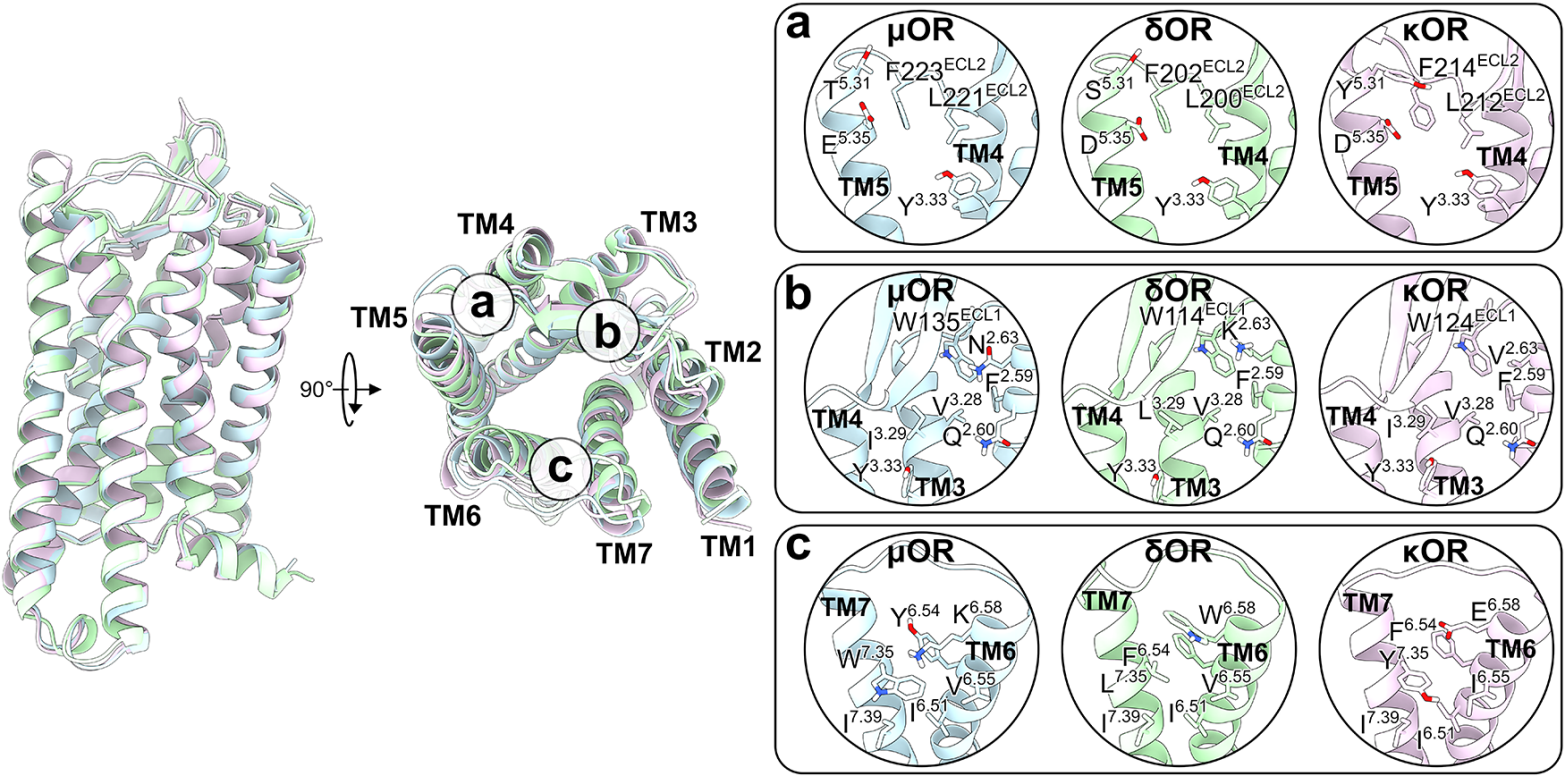
Identification of subtype-selective binding sites in the OR family. Regions a, b, and c represent subpockets around the orthosteric site of each receptor. OR structural features are observed based on the residue composition of each subpocket. The μOR, δOR, and κOR are represented as cartoons or sticks colored light blue, light green, and light purple, respectively. Oxygen, nitrogen, and polar hydrogen atoms are colored red, blue, and white, respectively.

Next, we found that region b corresponds to a hydrophobic subpocket formed by ECL1, TM2, and TM3 κOR residues, encompassing F^2.59^, V^2.63^, W124^ECL1^, and V^3.28^ residues (Figure 5, region b). Conversely, a substitution at position 2.63 in the κOR (V^2.63^) alters the hydrophobicity of region b in the other receptors, being replaced by a polar (N^2.63^) and positively charged (K^2.63^) residue in the μOR and δOR, respectively (Figure 5, region b). Similarly, region c is formed by TM6 and TM7 residues, with a high density of hydrophobic residues shaping a subpocket in the δOR, composed of I^6.51^, F^6.54^, V^6.55^, W^6.58^, L^7.35^, and I^7.39^ (Figure 5, region c). Two substitutions were identified in the other receptors that may alter the physicochemical properties of this region, where positions 6.58 and 7.35 in the δOR are respectively substituted by charged and aromatic residues in the μOR (K^6.58^ and W^7.35^) and κOR (E^6.58^ and Y^7.35^) (Figure 5, region c).

We further calculated the electrostatic influence zones and the Electrostatic Potential (ESP) in OR experimental structures (refer to Methods section). Electrostatic influence zones are volumes that encompass all electric field lines either emanating from or converging to a given atom, respectively termed electrophilic influence zone (EIZ) and nucleophilic influence zone (NIZ). These zones are unions of field lines bundles connecting pairs of negatively and positively charged sites.^85^ Our analysis reveals that the three regions previously identified by funnel-metadynamics are independent and confined EIZs in all three ORs, converging at the D^3.32^ residue (Figure 6). In the μOR, we observed a diffuse EIZ for W^7.35^, which extends through the receptor’s orthosteric site toward ECL2 residues, interacting with N^2.63^ and D218^ECL2^ residues (Figure S13). These EIZs also play a role in ligand stabilization at the OR orthosteric sites and extracellular vestibules, in which the three main binding states from our metadynamics results showed that morphine, buprenorphine, and NAQ form electric field lines with these zones in the μOR (Figure S14), δOR (Figure S15), and κOR (Figure S16). Notably, the distribution of hydrophobic and charged residues along the orthosteric sites toward extracellular vestibules of the ORs contributes to stabilizing S2 and S3 states of the ligands.

**Figure 6:**
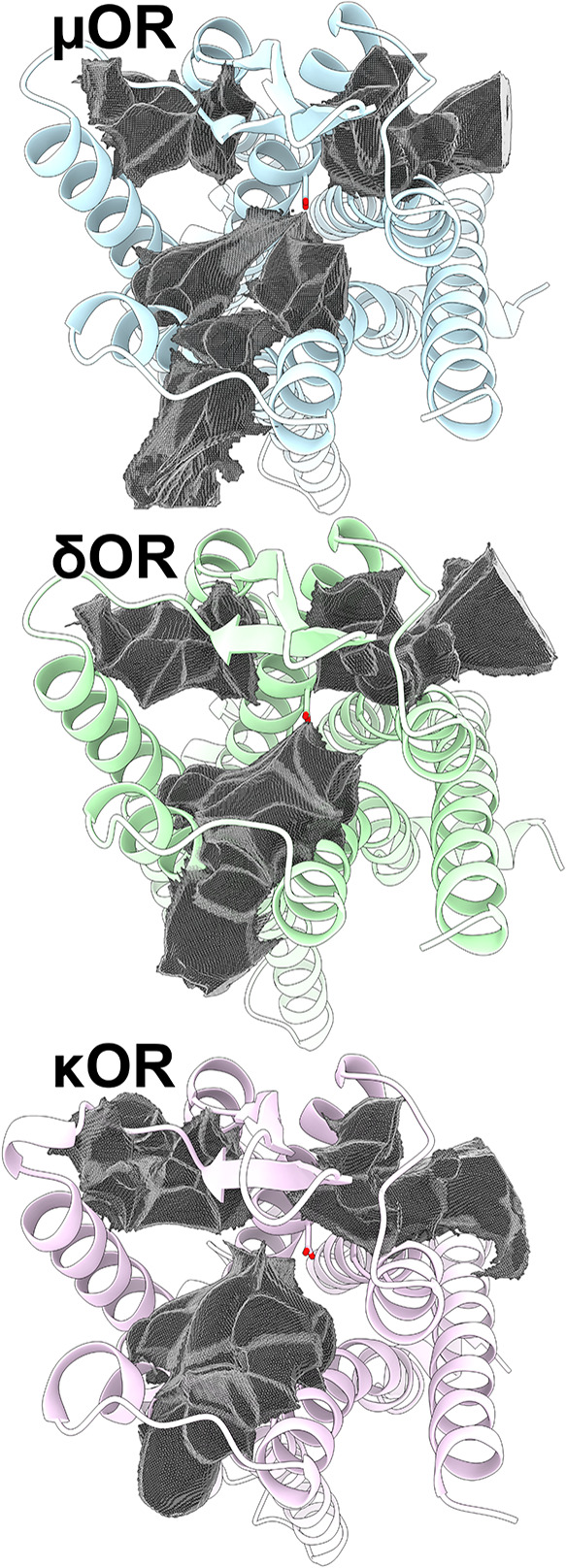
Identification of electrostatic influence zones (EIZs) in the OR family. The three regions previously identified by funnel-metadynamics represent independent EIZs. The μOR, δOR, and κOR are represented as cartoons or sticks colored light blue, light green, and light purple, respectively. D^3.32^ is shown as sticks using the corresponding colors, showing its side chain oxygens in red. The EIZ of the μOR residue W^7.35^ was omitted to facilitate the comparison between the receptors.

Finally, we calculated the ESP to reveal distinct charge distributions in OR orthosteric sites and extracellular vestibules. The ESP surface was converted into a 2D representation (Figure 7) by applying a nonlinear dimensionality reduction method that preserves the geometric topology of surfaces.^86^ The surface displays conserved charged regions at positions 3.32, 5.35, 5.39, and 6.52. The κOR has a more negatively charged orthosteric site compared to the δOR and μOR, which is likely due to the presence of E209^ECL2^ and E^6.58^ residues. Despite the presence of positively charged residues in the κOR's ECL2, such as K200^ECL2^ and R202^ECL2^, their accessibility to the solvent is hindered by surrounding negatively charged residues. In contrast, the δOR has positively charged residues located directly above its orthosteric site, which include K^2.63^, R192^ECL2^, and R291^ECL3^, while negatively charged residues are found at the extremity of its extracellular vestibule, such as E^2.67^, D193^ECL2^, D288^ECL3^, and D290^ECL3^. The μOR exhibits a balanced distribution of positively and negatively charged residues in its ECL2, comprising D218^ECL2^, K211^ECL2^, and R213^ECL2^, while presenting only one charged residue, E312^ECL3^, in its ECL3.

**Figure 7:**
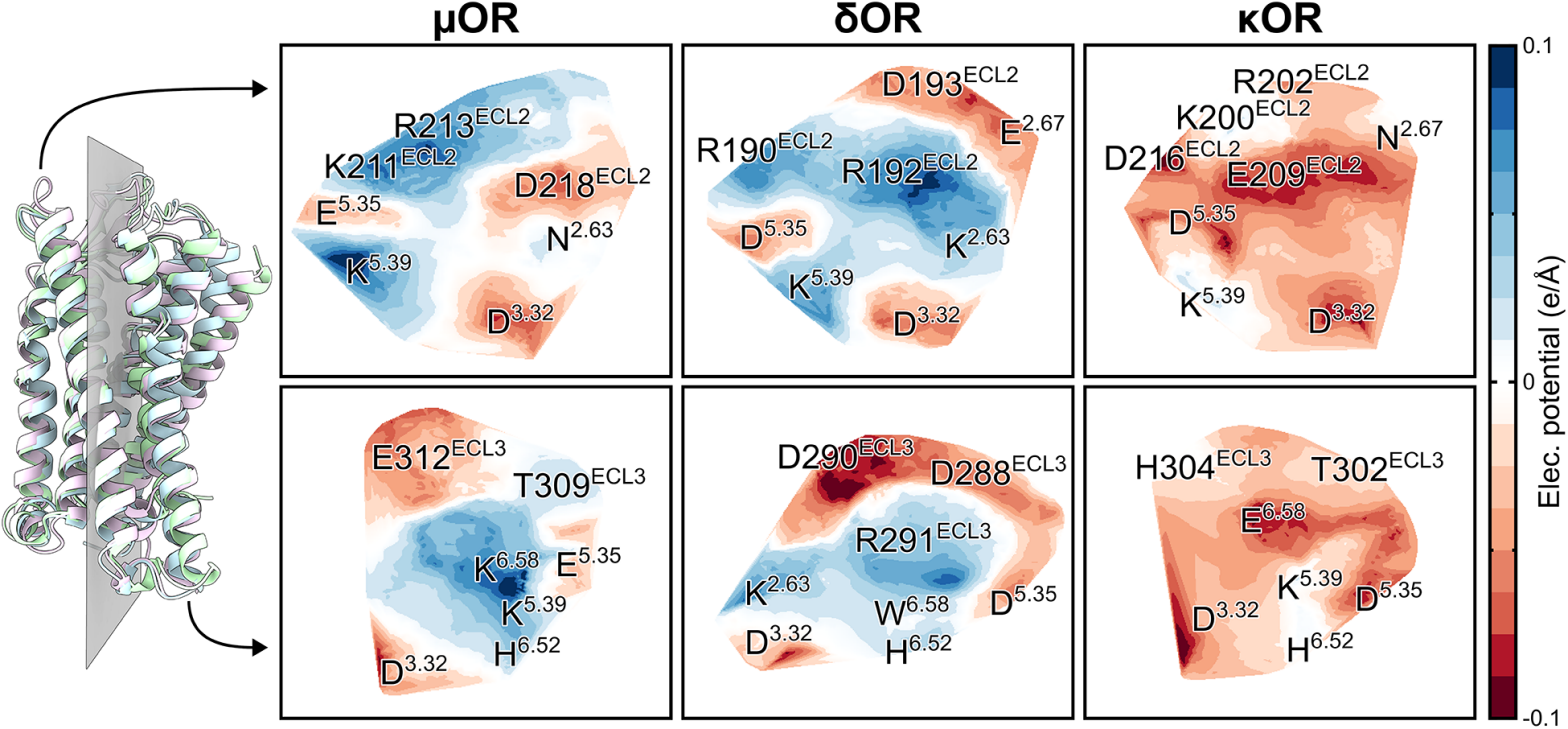
Electrostatic potential (ESP) surfaces of OR orthosteric sites and extracellular vestibules. μOR, δOR, and κOR are represented as cartoons or sticks colored light blue, light green, and light purple, respectively. We defined a plane, shown in gray, to determine ESP on each side of ORs. The left side of the receptors corresponds to the view of TM2–3 and TM5 residues (upper panels), while the right side corresponds to the view of TM6–7 residues (lower panels). Negatively and positively charged ESP surfaces are shown in red and blue, respectively, while neutral regions are shown in white. Surfaces were reshaped into a 2D representation by preserving their geometric topology.

## Discussion

Significant advances have been made in the study of GPCRs in the past 20 years, expanding our understanding of GPCR mechanisms of activation, signaling, and functional diversity,^87^ with remarkable implications in drug design for a rational and improved therapeutic targeting of these receptors.^88^ This has led to new approaches that have revolutionized the molecular pharmacology of these receptors in terms of ligand bias and functional selectivity, contributing to the investigation of more effective treatments with reduced side effects in pain management.^16–18^ Current advances strongly indicate that the receptor–ligand interface carries the information for intracellular signaling responses,^89^ where ligand-induced receptor conformations select preferred intracellular partners for diverse receptor functional responses.^90^ Therefore, identifying distinct receptor binding sites associated with specific pharmacological effects is crucial for obtaining safer signaling profiles in pain therapies.^34–36,38,54,91^ In this work, we explored receptor conformational dynamics and ligand binding, enhancing the spatial exploration of morphinan-scaffold ligands in receptor orthosteric sites and extracellular vestibules, to determine selective receptor-subtype binding regions that are part of electrostatic influence zones in the OR family. Our results present structural aspects that can be useful to advance the development of precise pharmacological therapies in these receptors.

We found that the μOR slightly increased the relative volume of its orthosteric site and extracellular vestibule, maintaining the receptor’s entrance frequently accessible. This is likely due to the electrostatic influence zone of W^7.35^ preventing interactions of ECL2 charged residues and the absence of complementary charged residues in ECL2 and ECL3 compared to δOR or κOR. This difference may represent a unique feature of the μOR compared to the other ORs, potentially explaining why ligands with large chemical groups, such as NAQ, selectively target the μOR.^32,74^ We found that the NAQ’s isoquinoline group accommodated into a hydrophobic subpocket between ECL2 and TM5 of the μOR, composed of leucine and phenylalanine. Although these residues are also present in the δOR and κOR, neighboring residues at positions 5.31 and 5.35, including charged ECL2 and ECL3 residues, may hinder NAQ binding to these receptors. Indeed, our metadynamics results for the μOR, mutated at these positions, show a reduction of approximately 2 kcal/mol compared to the wild-type receptor. These structural aspects may facilitate the selective binding of NAQ to the μOR compared to the other ORs.

Structure-based approaches targeting the distribution of charged residues near the subpocket formed by ECL2 and TM5 residues were crucial for developing new morphinan derivatives with selectivity for ORs. Uprety and collaborators^54^ synthesized selective μOR ligands by adding polar chemical groups to interact with E^5.35^ and ECL2 residues, such as D218^ECL2^ and T220^ECL2^. Our funnel-metadynamics results highlighted the importance of these residues in ligand-bound states S2 and S3, notably for stabilizing morphine through a polar network in S2. The engagement of μOR residues in ECL2 and TM5 in ligand interactions is associated with G protein-biased signaling,^54^ which is the case for NAQ and naltrexone derivatives.^32,74^ NAQ is also reported as a low-efficacy agonist at the μOR,^92–94^ supporting the ongoing discussion on the effectiveness of biased and low-efficacy agonists.^15–17,62^ As a μOR selective ligand with high affinity and low efficacy, NAQ antagonizes potent μOR agonists while displaying reduced secondary effects, showing promising applications for novel opioid use disorder therapeutics in withdrawal or dependence.^92–95^ According to our results, NAQ was the only ligand capable of accessing a vestibular region composed of ECL2 and TM5 residues in the lowest-energy state (S1), allowing us to describe the dynamic aspects of this region. These findings provide structural insights into μOR selectivity, extending alternatives for exploring biased or low-efficacy pharmacological profiles. Furthermore, a new subpocket for partial agonism at the μOR, formed by TM1, TM2, and TM7 residues, has been recently discovered as essential for stabilizing hydrophobic interactions with the partial agonist and G protein-biased mitragynine pseudoindoxyl.^38^ We identified the same subpocket occupied by buprenorphine, another partial agonist at the μOR,^75^ in μOR S2 and S3 states. These results align with previous work using distinct MD parameters.^96^

Regarding the δOR, we identified a hydrophobic subpocket between TM6 and TM7 as essential for stabilizing the lowest-energy state of all ligands, including intermediate states (S2 and S3) of morphine and buprenorphine. While hydrophobic residues shape the pocket in the δOR, charged or aromatic residues in the μOR and κOR represent a structural distinction in these receptors (Figure 5, region c). Targeting this pocket has led to the development of selective small molecules^42,43,45,46,52,97^ and peptides^98^ at the δOR. Mutagenesis scanning studies have identified the subpocket residues W^6.58^ ^99–101^ and L^7.35^ ^101–103^ as crucial in ligand binding and selectivity at the δOR. In addition, the substitution L^7.35^W impaired ligand binding to the δOR, likely due to changes in the shape of the receptor’s subpocket.^101^ We identified both residues as essential in all states of the ligands examined in this study, emphasizing their role in ligand interactions. Conversely, ECL3 residues, such as R291^ECL3^, have been reported to participate in peptide selectivity.^98,102,103^ Our results found R291^ECL3^ stabilizing interactions with morphine in the S3 state and buprenorphine in S2 and S3 states. R291^ECL3^ also participated in a salt bridge with D193^ECL2^, facilitating the lid formation above the δOR orthosteric site. Although further studies are required, our results indicate the involvement of R291^ECL3^ in some ligand intermediate states, potentially participating in the initial steps of ligand recognition and accommodation before binding into the δOR orthosteric site.

In this study, we captured unique structural aspects of the κOR compared to other ORs, notably by forming a lid that covers its orthosteric site due to a salt bridge between R202^ECL2^ and E^6.58^. Our metadynamics results suggest that these motions cost approximately 6.45 kcal/mol and are affected when the receptor is bound to buprenorphine or NAQ. Although experimental structures did not capture the salt bridge observed in our results, a lid has been reported in κOR-bound structures with the inverse agonist JDTic^59^ and the G protein-biased agonist nalfurafine.^60^ Additionally, even if it should always be considered that the force field may overestimate the equilibrium of these movements, most of the available experimental structures did not model side chains of some ECL2 residues,^59,60,104,105^ indicating a flexible region. These aspects justify the inclusion of CV_bridge_ as a third CV in our funnel-metadynamics calculations to successfully predict protein–ligand free energy of binding at the κOR (refer to Methods section). A similar approach was reported by Mattedi and collaborators,^71^ which suggested that salt bridges at the entrance of the orthosteric site in adenosine receptors play a role in receptor dynamics and ligand interaction. Furthermore, the ligand-induced impairment in lid formation emphasizes the relevance of ECL2 and ECL3 interactions in the receptor’s dynamics. This aligns with a previous report that describes a network of residues in ECL2-ECL3 and TM6–TM7 that modulate the activity of the κOR, including E209^ECL2^, L212^ECL2^, E^6.58^, L^7.32^, and Y^7.36^.^105^ Our results identified L^7.32^ as participating in S2 and S3 states of all three ligands, while the phenol groups of Y^7.35^ and Y^7.36^ formed hydrogen bonds with morphine in S2 and S3 states. E209^ECL2^ and S221^ECL2^ were reported as essential for ligand stabilization, with the latter likely increasing the nalfurafine residence time at the κOR.^60^ Similar findings have been reported in dopamine^106^ and serotonin^107^ receptors. Our results suggest that ECL2 residues play a role in stabilizing buprenorphine and NAQ in the κOR orthosteric site in the lowest-energy state (S1) identified in our work. We also found that W124^ECL1^ was critical for stabilizing buprenorphine in all states of our funnel-metadynamics results, reinforcing its role in ligand efficacy.^104^ Overall, our results reveal potential κOR selective binding sites in ECL1 and ECL2 that likely modulate ligand residence time, including W124^ECL1^, E209^ECL2^, C210^ECL2^, S221^ECL2^, and L212^ECL2^.

We identified a hydrophobic subpocket in the κOR, formed by ECL1, TM2, and TM3 residues, that is occupied by buprenorphine in all three main states, corresponding to biased signaling at the κOR.^54^ Mutations in this subpocket affected ligand potency or reduced β-arrestin recruitment.^60,108^ Notably, κOR antagonists^57,59^ or agonists^58,60,104^ have been reported to occupy this hydrophobic pocket. Our results indicate that buprenorphine interacts with this subpocket and engages in strong TM7 interactions through its 2-hydroxy-3,3-dimethylbutan-2-yl and *N*-methylcyclopropyl group, respectively, potentially explaining its antagonism at the κOR. In agreement with this, norbuprenorphine, an active metabolite of buprenorphine missing the *N*-methylcyclopropyl group and likely presenting reduced TM7 interactions, exhibits moderate partial agonism at the κOR.^109^ Conversely, morphinan-scaffold ligands containing the *N*-methylcyclopropyl group can also induce agonist effects at the κOR, as observed in the partial agonist nalfurafine (PDB ID: 7YIT)^60^ and the agonist MP1104 (PDB ID: 6B73).^58^ When comparing these complexes with the S1 state of buprenorphine from our metadynamics results, we observe differences in how this hydrophobic subpocket in the κOR is accessed (Figure S17). While buprenorphine penetrates deeper in the hydrophobic subpocket, interacting with residues such as V^2.63^, nalfurafine and MP1104 interact with T^2.56^. Furthermore, the chemical differences between the furan ether of nalfurafine and the iodobenzene of MP1104 compared to the 2-hydroxy-3,3-dimethylbutan-2-yl of buprenorphine may also play a role in inducing distinct pharmacological profiles at the κOR. Our results indicate that subtle differences in chemical groups when accessing this subpocket can affect κOR signaling.

Recent work has shown that receptor residue contributions in ligand binding are critical for understanding how GPCRs interpret extracellular information into signaling responses.^89^ This reinforces the “message-address” functioning of GPCRs, including ORs, in which a set of residues is involved in ligand efficacy (message) while others contribute to ligand selectivity (address).^110–113^ These findings are paving the way for the development of new therapies in pain management with tuned pharmacological profiles to reduce side effects.^16,17,62^ The identification of the OR family experimental structures bound to their respective endogenous opioid peptides has expanded the “message-address” hypothesis by confirming the presence of a common hydrophobic pocket at the bottom of OR orthosteric sites, which is related to the “message region” and by revealing the role of OR subpockets and extracellular vestibules as the “address region” in receptor selectivity.^114^ Using computational approaches, we support the existence of these regions by investigating how ligands bind to ORs. We highlight the existence of a conserved ligand-binding pocket for morphinan-scaffold molecules located at the bottom of OR orthosteric sites, which connects with electrostatic influence zones in orthosteric subpockets or ECL residues.

These subtype-selective binding regions have unique residue compositions that can be accessed to explore diverse pharmacological profiles at ORs (Figure 5). For example, ligands that interact at position 5.31 in region a can selectively target these receptors. In contrast, ligands with positively charged groups accessing this same region may show a preference for the μOR (D218^ECL2^) and κOR (E209^ECL2^) over the δOR (R192^ECL2^), as previously shown experimentally.^54^ Furthermore, ligands that interact with region b and form polar interactions at position 2.63 could dual-target the μOR (N^2.63^) and δOR (K^2.63^) instead of the κOR (V^2.63^). Similarly, polar interactions in region c at position 6.58 can target the μOR (K^6.58^) and κOR (E^6.58^) rather than the δOR (W^6.58^). Previous research showed that position 6.58 plays a role in the selectivity of endogenous opioid peptides in the OR family.^114^ Distinctions in physicochemical properties of these residues in these regions can facilitate the design of single-target ligands. Moreover, these binding sites indicate entry and egress pathways that can provide distinct pharmacological profiles by modulating ligand residence times.^115^ All these features can also be explored by aligning biophysical and machine learning techniques with experiments to advance research in the field.^116^ Altogether, our findings highlight OR binding regions that can be rationally targeted in the development of single-, dual- or multitargeting ligands,^117–119^ also accounting for their residence times,^115^ to aid structure-based drug design and discovery campaigns toward safer alternatives in pain management.

## Conclusions

In the present study, we combined computational techniques to explore conformational dynamics and understand how morphinan-scaffold ligands bind to ORs, accessing their orthosteric sites and extracellular vestibules. The utilization of metadynamics was crucial to determining these structural aspects from an energetic perspective. Metadynamics has also accurately predicted ligand binding affinities to each OR, describing the residue contributions in distinct ligand-bound states. Our work emphasizes the importance of potential selective binding regions in OR orthosteric subpockets and extracellular vestibules, highlighting the relevant role of ECL residues. These regions also provide opportunities for exploring OR polypharmacology, developing orthosteric or allosteric compounds that promiscuously or selectively target these receptors, thereby extending the therapeutic window of existing opioid compounds. Although our work focused on morphinan-scaffold ligands, including other classes of ligands would expand our understanding of structural and functional selectivity within the OR family. The approach used in this work can serve as a framework for classifying ligands based on their binding affinities and identifying functionally relevant binding sites in GPCRs, bringing insights into the rational design of drugs with improved pharmacological profiles.

## Methods

### Molecule Preparation

The cryo-EM structures of the human μOR, δOR, and κOR in the active state were retrieved from the Protein Data Bank (https://www.rcsb.org)^120^ under the respective codes 8EF6,^34^ 8F7S,^114^ and 8F7W.^114^ We preserved all mutations designed for thermostability in the δOR structure,^114^ as they were located away from the receptor’s orthosteric site and extracellular vestibules. μOR double mutants T^5.31^S/E^5.35^D and T^5.31^Y/E^5.35^D, respectively named μOR-SD and μOR-YD, were generated using the CHARMM-GUI web server. The protonation state of the receptor residues was calculated at a physiological pH of 7.4 using the CHARMM-GUI web server.^121^ All acidic and basic residues were set to their charged states, while all histidines were set as neutral with hydrogen atoms bound to the nitrogen δ. Morphine, buprenorphine, and NAQ structures were built using the R.E.D server,^122^ maintaining their amino group protonated to reproduce physiological pH conditions. Complexes were obtained by aligning the Cα atoms of the receptors to the μOR and the ligand morphinan-scaffold atoms of buprenorphine or NAQ to morphine in the μOR/morphine complex. Although buprenorphine acts as an antagonist at the δOR and κOR, we decided to use the active state for all receptors to have a common structural framework for all ligands as a starting point in all simulations. Additionally, our work focused on allowing the ligands to explore OR orthosteric sites and extracellular vestibules, whose shapes are structurally conserved in active and inactive states.^123^

### MD Simulations

We used the CHARMM-GUI web server^124^ to prepare all systems. Disulfide bonds were patched between C^3.25^ and the ECL2 residues C219^ECL2^, C198^ECL2^, or C210^ECL2^ in the μOR, δOR, and κOR, respectively. Protein, water, and ion parameters were generated under the Amber ff19SB force field,^125^ while the topology for phosphatidylcholine (POPC) was obtained using Lipid21 parameters.^126^ Ligand force field parameters were obtained from the AMBER force field 2 (GAFF2),^127^ updating their charges using HF/6-31G­(d) restrained electrostatic potential (RESP-A1) charges implemented in the R.E.D server.^122^ Each complex was placed in a hexagonal box and embedded in a POPC bilayer to obtain an initial system size of approximately 9.5 × 9.5 × 13 nm in XYZ dimensions. Subsequently, each system was solvated with TIP3P water models and then neutralized with potassium and chloride ions to reach a 0.15 M concentration. Initial input parameters for MD simulations were prepared to be compatible with GROMACS v.2022.2.^128^ A minimization step was conducted using the Steepest Descent algorithm until an energy gradient below 100 kJ/mol/nm was reached. Furthermore, successive equilibration steps were performed according to the parameters specified in our previous work.^96^ Briefly, the system’s velocities were randomly generated according to a Maxwell–Boltzmann distribution at 300 K using the V-Rescale thermostat.^129^ The Parrinello–Rahman barostat^130^ monitored the system pressure at 1 bar using a semi-isotropic coupling. Position restraint forces applied on the receptor backbone, side chain, ligand non-hydrogen, and lipid head atoms were progressively reduced to zero. Production was performed in four independent replicas lasting 1.0 μs for unbound systems and one replica of 1.0 μs for ligand-bound systems, with frames collected every 20 ps. Nonbonded interactions were calculated up to 0.9 nm, while long-range electrostatic interactions were calculated within a 0.9 nm cutoff using the particle-mesh Ewald (PME) method^131^ with dispersion correction for energy. Representative MD simulation boxes for unbound and ligand-bound systems are shown in Figure S18.

### Metadynamics

Metadynamics simulations were conducted to estimate the lid formation in the κOR and ligand binding affinity, including intermediate states, in all three ORs using GROMACS v2019.6 ^128^ patched with PLUMED v2.8.1.^132^ MD parameters were set as for classical MD simulations, described above, employing well-tempered metadynamics^78,79^ to all simulations. The starting point of each complex for metadynamics simulations was obtained after 0.5 μs of classical MD simulations.

For exploring the lid formation in the κOR, we defined CV_bridge_ as the distance between the CZ atom of R202^ECL2^ and the CD atom of E^6.58^. We applied an upper wall at 2.4 nm using a quadratic repulsive potential force of 1500 kJ/mol/nm^2^ to prevent large exploration of the CV_bridge_. We then performed an initial metadynamics simulation to extract 20 representative structures for each 0.1 nm window along CV_bridge_ to be used as starting points for multiple walkers.^133^ Gaussian hills parameters, height and width, were respectively set to 3 kJ/mol and 0.033 nm, with an initial bias factor of 20 applied at every 1000 steps. During the production phase, Gaussian hills height was rescaled to 1 kJ/mol while the bias factor was rescaled to 10. Subsequently, 300 ns were run for each complex. Free energies were calculated by summing the Gaussians using the *sum_hills* function from the PLUMED plugin.^132^ We obtained free energy values over the lowest-energy well compared to the region between 1.4 and 1.6 nm, corresponding to the experimental structure, in CV_bridge_ by calculating the integral and the mean, respectively. Free energy differences between the wells identified in the κOR and the plateau around 1.3 nm were calculated using the integral and the mean, respectively. For the κOR/NAQ complex, we obtained free energies using the integral over the two wells. Mean and standard deviation of free energy values for CV_bridge_ were calculated every 1 ns using the last 50 ns of the simulated time.

To predict ligand binding affinity, we applied funnel-metadynamics, a technique that imposes funnel-shaped constraints to limit ligand exploration in the solute, thereby enhancing simulation convergence.^80^ We defined the distance between the Cα carbon of W^6.48^ and the nearest atoms of the ligand’s center of mass to determine the projection onto the *Z*-axis (Z-projection) and the XY-plane (XY-projection), respectively, perpendicular and parallel to the membrane.^69,71^ For the κOR, we included CV_bridge_ as a third CV. A funnel-like restraint^69,134^ was applied according to eq 1:

1 $$r=h\times \frac{1}{1+e^{s\left(z-z_{0}\right)}}+b$$

where *r* denotes the XY-projection, *h* = 2.2 nm is the funnel width, *s* = 1.2/nm is the funnel steepness, *z* is the Z-projection, *z*_0_ = 4 nm is the inflection point of the funnel, and *b* = 0.3 nm is the minimum funnel width. Upper and lower walls were respectively set at 6 and 0.5 nm in the Z-projection with a repulsive potential force of 1500 kJ/mol/nm^2^, thus preventing the ligand from crossing the funnel boundaries.

We performed an initial metadynamics simulation to extract 20 representative structures for each 0.3 nm window along the Z-projection, serving as starting points for multiple walkers.^133^ Gaussian hills parameters, height and width, were respectively set to 2 kJ/mol and 0.1 nm for XY- and Z-projections. For the κOR, CV_bridge_ was included using the parameters described above. An initial bias factor of 20 was applied at every 1000 steps in all complexes. During the production phase, Gaussian hills height was rescaled to 1 kJ/mol in μOR and δOR complexes, and 1.5 kJ/mol in κOR complexes. The bias factor was set to 10 in all complexes. Free energies of binding were calculated using *sum_hills*, as described above, correcting the loss of translational and rotational freedom of the ligand in the unbound state imposed by funnel boundaries, according to the protein–ligand free energy of binding eq 2:

2 $$\Delta G=-\kappa_{B}T\ln\left(K_{b}C^{0}\right)$$

where κ_*B*_ is the Boltzmann constant, *T* is the temperature of the system, and *C* ^0^ = 1/1.66/nm^3^ is the standard concentration. The binding constant *K*_*b*_, eq 3, is defined as

3 $$K_{b}={\int_{\mathrm{bound}}dze}^{[\frac{-\left(W_{\left(z\right)}-W_{ref}\right)}{\kappa_{B}T}]}\pi R_{cyl}^{2}$$

where *z* is the coordinate along the Z-projection, with *W*_*(z)*_ and *W*_*ref*_ corresponding to the free energy in the bound and unbound states, respectively. We obtained free energy values over the bound and unbound states by calculating the integral and the mean, respectively. $\pi R_{cyl}^{2}$ is the surface of the cylinder determined by its radius (*R*), accounting for the volume correction for the funnel restraint potentials.^80^ We defined the unbound region from 5.4 to 5.5 nm along the Z-projection, resulting in a correction value of 0.2615 kcal/mol for all complexes. Mean and standard deviation of free energy values for ligand-bound states were calculated every 1 ns using the last 200 ns of the simulated time.

### Electrostatics and Charge Density Calculations

The atomic charges *q_i_* of OR atoms were retrieved from the ELMAM2 database of multipolar atoms.^135^ We applied electroneutrality constraints to each residue while assigning a charge of +1 e to lysines and arginines, and −1 e to glutamic and aspartic acids. The X–H bond lengths were elongated according to standard neutron diffraction distances.^136^ These parameters were used for calculating electrostatic influence zones and the ESP for each OR experimental structure. The ESP was computed using a point charge model implemented in the VMoPro module of the MoProSuite software.^137^ As used in biomolecular force fields, we applied the dielectric value (ε) dependent on the distance ε = *r*ε_0_, where *r* is the distance to the atom.^138^ Therefore, the ESP was computed as a summation over each atom *i*, with eq 4:

4 $$V\left(r\right)=\underset{i}{\sum }\frac{q_{i}}{4\pi \varepsilon r}=\underset{i}{\sum }\frac{q_{i}}{4\pi \varepsilon_{0}r^{2}}$$

To represent the ESP in OR orthosteric sites and extracellular vestibules, we employed Epock^77^ to cover these regions with spheres of 1.0 and 1.2 nm radii using a grid spacing of 0.05 nm and a probe radius of 1.4 nm. A seed sphere with a radius of 0.4 nm was placed between both spheres, using a 1.0 nm contiguous cutoff. We then preserved the surface points using a homemade Python script, transferring the ESP values to these points. The steps of this strategy are summarized in Figure S19. The proximity of the side chains of arginines R192^ECL2^ and R291^ECL3^ formed a hole in the δOR extracellular vestibule. To address this issue, we calculated the surface points on each side of the three ORs, preserving the points corresponding to their orthosteric sites and extracellular vestibules. For ESP calculations of the δOR, we removed the side chain of the opposite arginine while maintaining a charge of +1 e on their Cα atoms. We then performed a nonlinear dimensionality reduction of these surface points using the Isomap function,^86^ implemented in scikit-learn, to obtain a 2D space representation of the ESP of OR orthosteric sites and extracellular vestibules.

Electrostatic influence zones were computed numerically in MoProViewer^137^ using regular 3D grids with a uniform sampling of 0.05 Å of the ESP, following the approach described previously. The ESP gradient was computed at each grid point and followed in both directions in 0.05 Å steps to define electric field lines. If a line originates from or terminates at the considered site (e.g., a hydrogen atom in the case of an EIZ), all points along that line are flagged as belonging to the influence zone. This procedure defines a binary implicit function, the 0.5 iso-surface of which represents the boundary of the electrostatic influence zone.

### Structural Analysis

The minimum distance between R202^ECL2^ and E^6.58^ at the κOR and between D193^ECL2^ and R291^ECL3^ at the δOR, including the distance of CV_bridge_, from classical MD simulations was calculated using the GROMACS plugin *mindist*,^128^ defining the desired atoms as index groups. RMSD calculations were performed using VMD^139^ to compare the morphine-bound μOR experimental structure^34^ with the S1 state obtained from metadynamics and obtain the mean and standard deviation, considering the non-hydrogen atoms of morphine after aligning the backbone atoms of the receptor. The percentage of contacts was calculated considering atomic distances within a 0.4 nm cutoff using VMD scripting^139^ to determine the residue contribution of each complex. Ensembles of conformations for each ligand intermediate state were obtained by selecting frames within their respective wells in XY- and Z-projections. The percentages of contacts were also used to reveal receptor binding sites from concatenated metadynamics trajectories. Volume calculations were performed using Epock,^77^ covering the orthosteric sites and extracellular vestibules of the receptors with spheres with radii of 0.6 and 0.9 nm, respectively, using a grid spacing of 0.05 nm and a probe radius of 1.4 nm. A seed sphere with a radius of 0.4 nm was placed between both spheres, using a 0.2 nm contiguous cutoff. Receptor relative volumes were obtained by dividing the volume of each frame by the respective volume obtained from the experimental structures used in this work. All structural illustrations were generated with ChimeraX v1.9 ^140^ or MoProViewer,^137^ 2D representations of ligands were generated with Ketcher (https://lifescience.opensource.epam.com/ketcher/index.html), and all graphs were built using Gnuplot v6.0 (https://www.gnuplot.info).

## Data and Software Availability

All input files necessary to reproduce our classical MD and funnel-metadynamics simulations, including treated trajectories and metadynamics outputs (HILLS and COLVAR files), are freely available at http://10.5281/zenodo.15862810.

## Supplementary Material



## Notes

2D representation of ligands (Figure S1); time-dependent OR salt bridges (Figure S2); structural aspects of the κOR related to the collective variable CV_bridge_ (Figure S3); time-dependent free energy values and profiles of CV_bridge_ (Figure S4); time-dependent free energy values (Figure S5); profiles of funnel-metadynamics (Figure S6); percentages of contacts from funnel-metadynamics of μOR (Figure S7), δOR (Figure S8), and κOR bound to ligands (Figure S9); identification of OR binding sites (Figure S10); free-energy values (Figure S11) and free-energy landscapes of funnel-metadynamics of the μOR mutants bound to NAQ (Figure S12); electrostatic influence zones of the μOR residue W^7.35^ (Figure S13); electrostatic influence zones of ligands bound to μOR (Figure S14), δOR (Figure S15), and κOR (Figure S16); structural comparison of buprenorphine to other κOR modulators (Figure S17); representation of MD simulation boxes for unbound and ligand-bound systems (Figure S18); and strategy to represent electrostatic potentials of OR orthosteric sites in 2D (Figure S19) (PDF)
