# Supplementary material for: Identification of Subtype-Selective Binding Sites in the Opioid Receptor Family
Source: J Chem Inf Model. 2026 Apr 3;66(8):4717–33. doi: 10.1021/acs.jcim.5c02403 (PMC13126641; doi:10.1021/acs.jcim.5c02403)
Supplement: Supplementary file 1 [file ci5c02403_si_001.pdf]

Supplementary Information

Identification of subtype-selective binding sites in the opioid receptor family

*Antoniel A. S. Gomes[1,2,3], Benoît Guillot[4], Christian Jelsch[4], and Jesús Giraldo[1,2,3]**

[1]Laboratory of Molecular Neuropharmacology and Bioinformatics, Unitat de Bioestadística and Institut de Neurociències, Universitat Autònoma de Barcelona, 08193 Bellaterra, Spain

[2]Unitat de Neurociència Traslacional, Parc Taulí Hospital Universitari, Institut d'Investigació i Innovació Parc Taulí (I3PT), Institut de Neurociències, Universitat Autònoma de Barcelona, 08193 Bellaterra, Spain

[3]Instituto de Salud Carlos III, Centro de Investigación Biomédica en Red de Salud Mental (CIBERSAM), 28029 Madrid, Spain

[4]CRM[2], CNRS UMR 7036, Faculté des Sciences et Technologies, Université de Lorraine, 54000 Nancy, France

*: corresponding author: Jesus.Giraldo@uab.cat

This document contains supplementary figures S1 to S19 and Table S1.

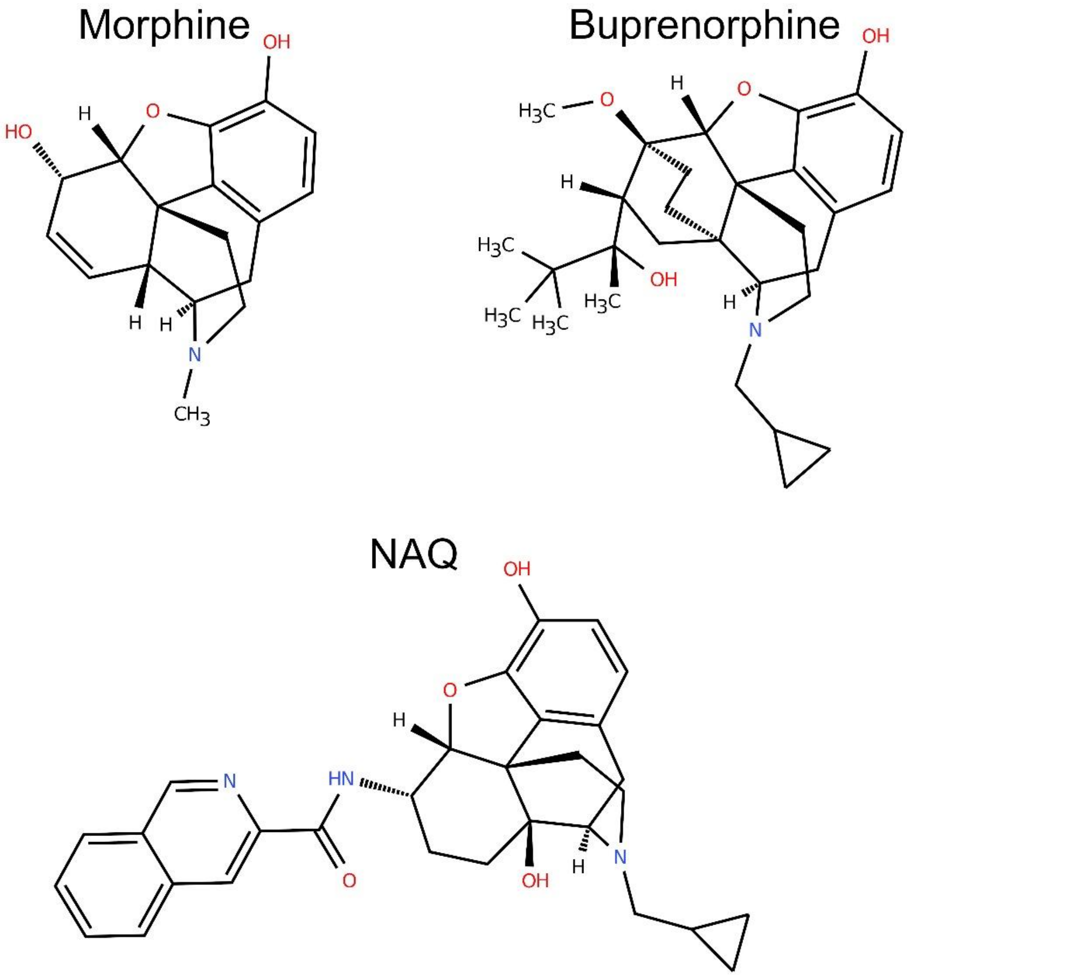

Figure S1. 2D representation of morphine, buprenorphine, and NAQ. Oxygen and nitrogen atoms are colored red and blue, respectively.

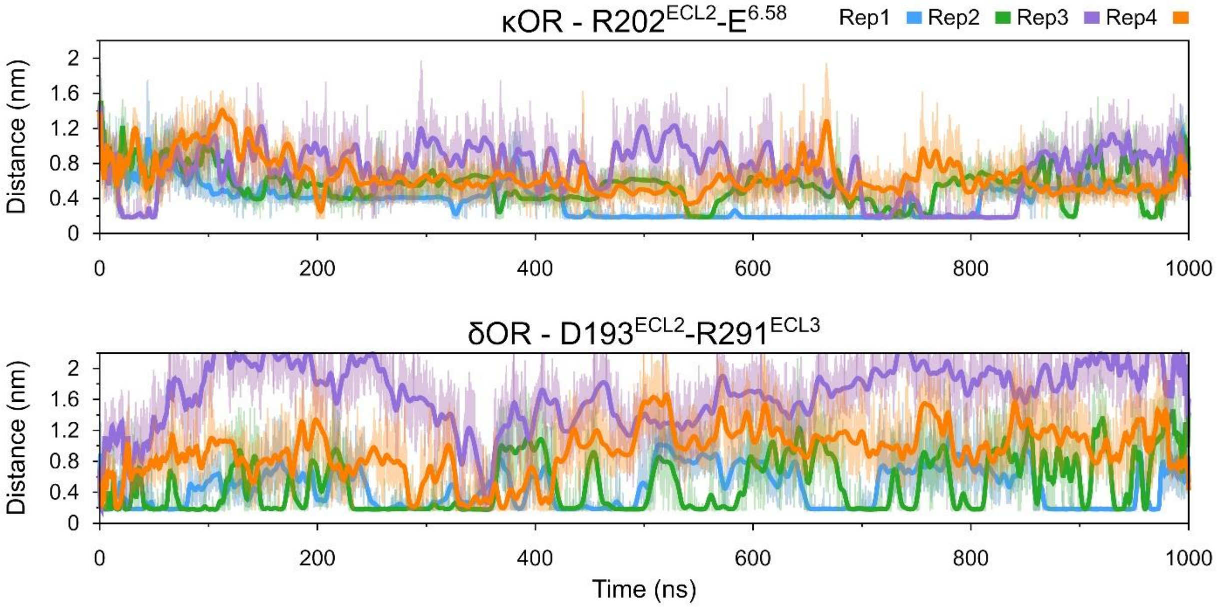

Figure S2. Time-evolution of κOR and δOR residues forming salt bridges during MD simulations. We show (upper panel) the minimum distance between R202^{ECL2} and E$^{6.58}$ in the κOR and (lower panel) between D193^{ECL2} and R291^{ECL3} in the δOR, in which salt bridges are observed in distances below 0.3 nm.

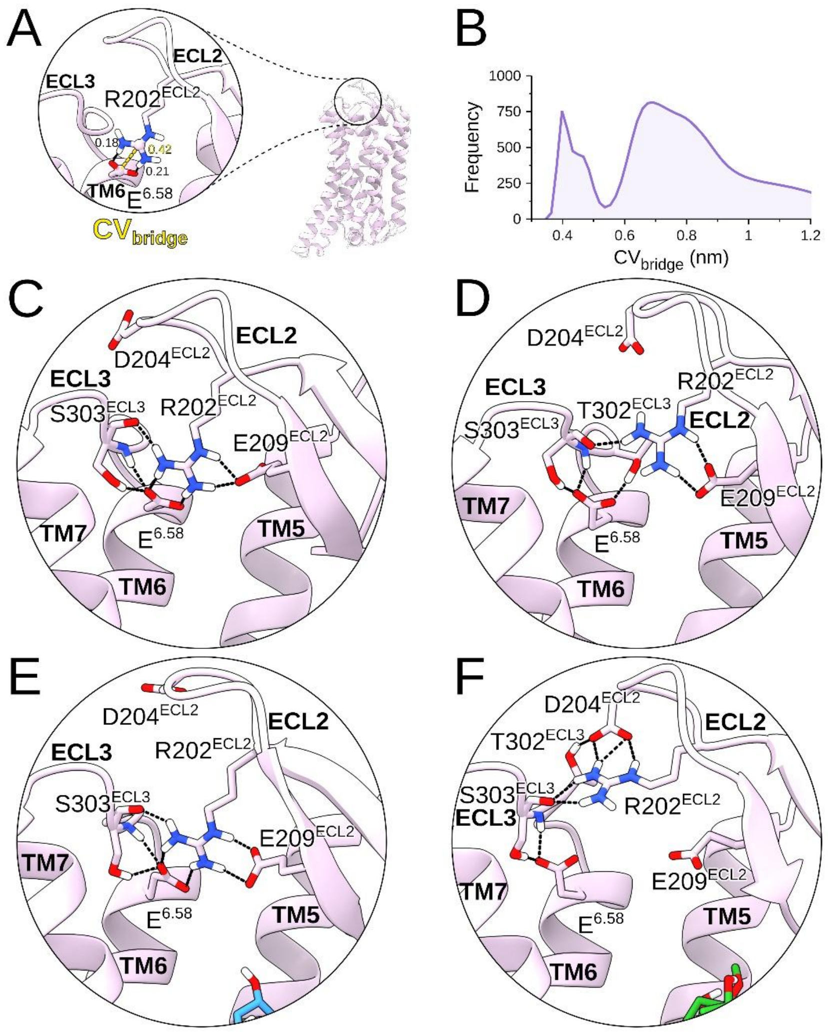

Figure S3. Structural aspects of the formation of a lid in the κOR. (A) The collective variable CV_{bridge} was defined to explore the distance between the CZ atom of $R202^{ECL2}$ and the CD atom of $E^{6.58}$. (B) Frequency distribution of CV_{bridge} from the four independent replicas of classical MD simulations of the unbound receptor shows two high-frequency regions at around 0.41 and 0.65 nm. These same regions were also identified by metadynamics, in which structural aspects corresponding to the distances at (C) 0.40 nm and (D) 0.70 nm are shown. Similar conformations were obtained by funnel-metadynamics results of the receptor bound to (E) morphine and (F) buprenorphine. The κOR is represented as light purple cartoons or sticks, while morphine and buprenorphine are shown as blue and green sticks, respectively.

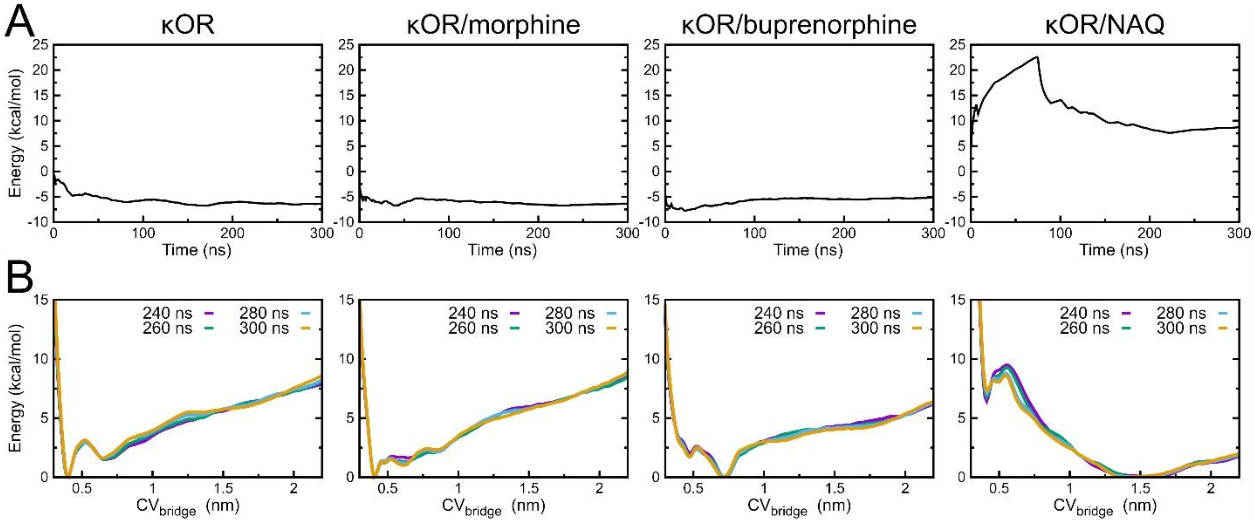

Figure S4. Time-evolution of the computed free energy values of CV_{bridge} using metadynamics. (A) Changes in free energy values as a function of the sampling time (every 1 ns). Positive values in the κOR/NAQ complex show the shift of the minimum to around 1.5 nm. (B) Changes in free energy profiles as a function of the sampling time (every 20 ns) show the convergence of metadynamics simulations for the κOR unbound and bound to morphine, buprenorphine, and NAQ.

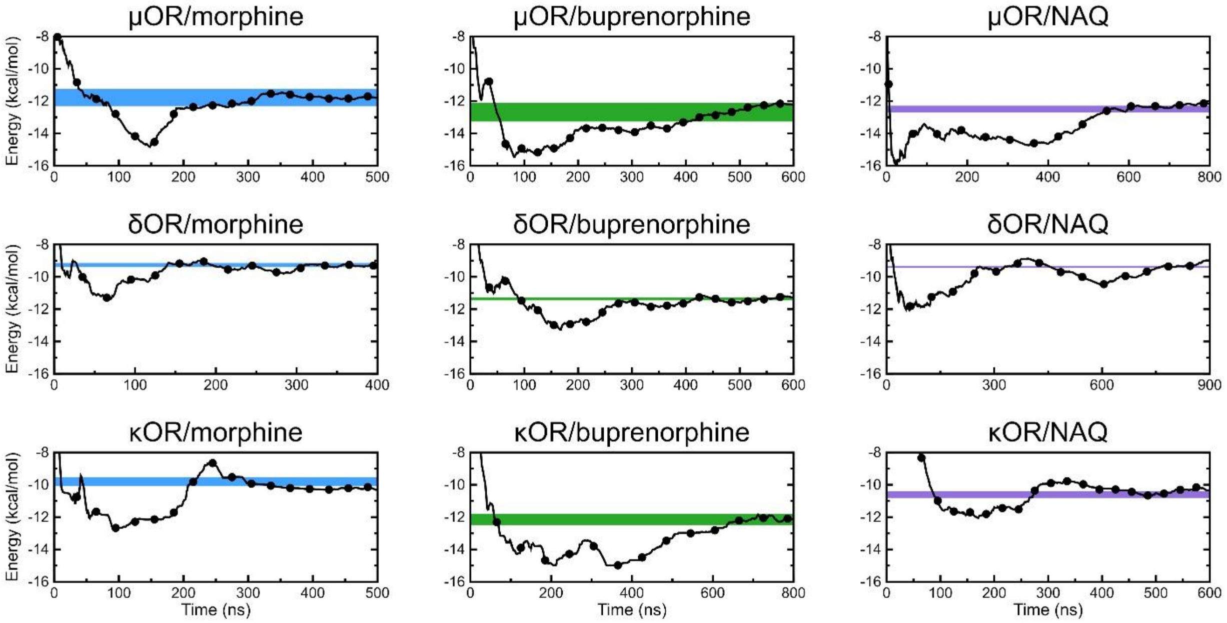

Figure S5. Time-evolution of the computed free energy values using funnel-metadynamics. Changes in free energy values as a function of the sampling time (every 1 ns) show the convergence of metadynamics simulations for each OR bound to morphine, buprenorphine, and NAQ. Horizontal lines indicate the experimental range of free energies for ligand binding to ORs.

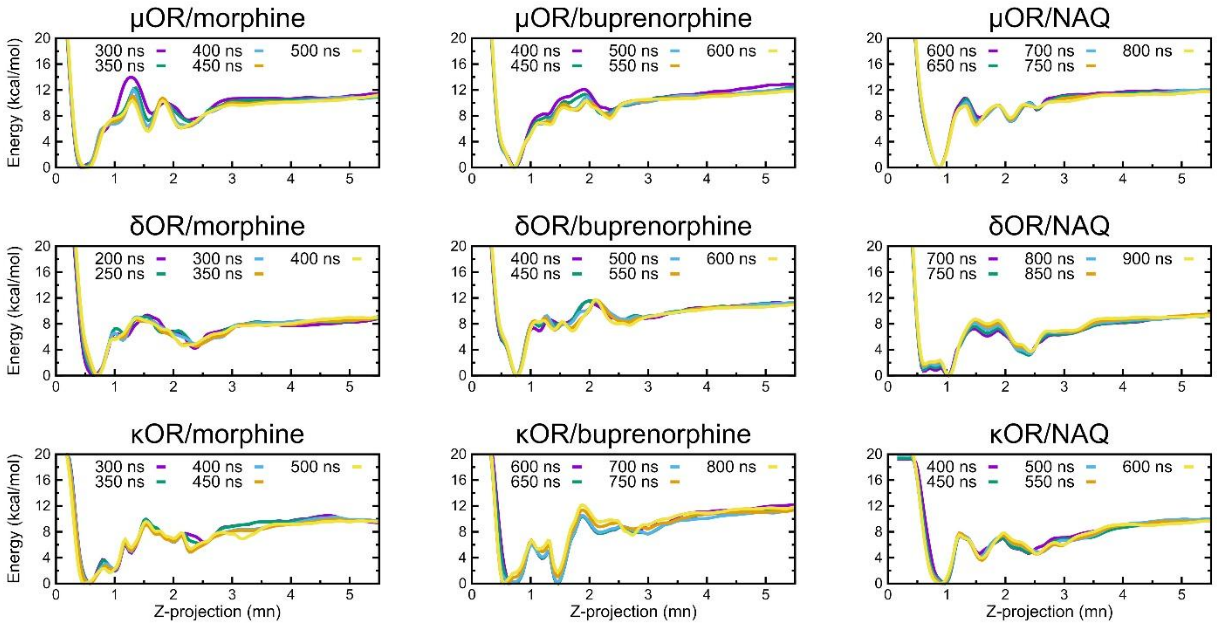

Figure S6. Time-evolution of the computed binding free energy profiles using funnel-metadynamics. Free energy profiles as a function of the sampling time (every 50 ns) show the convergence of metadynamics simulations considering the Z-projection for each OR bound to morphine, buprenorphine, and NAQ.

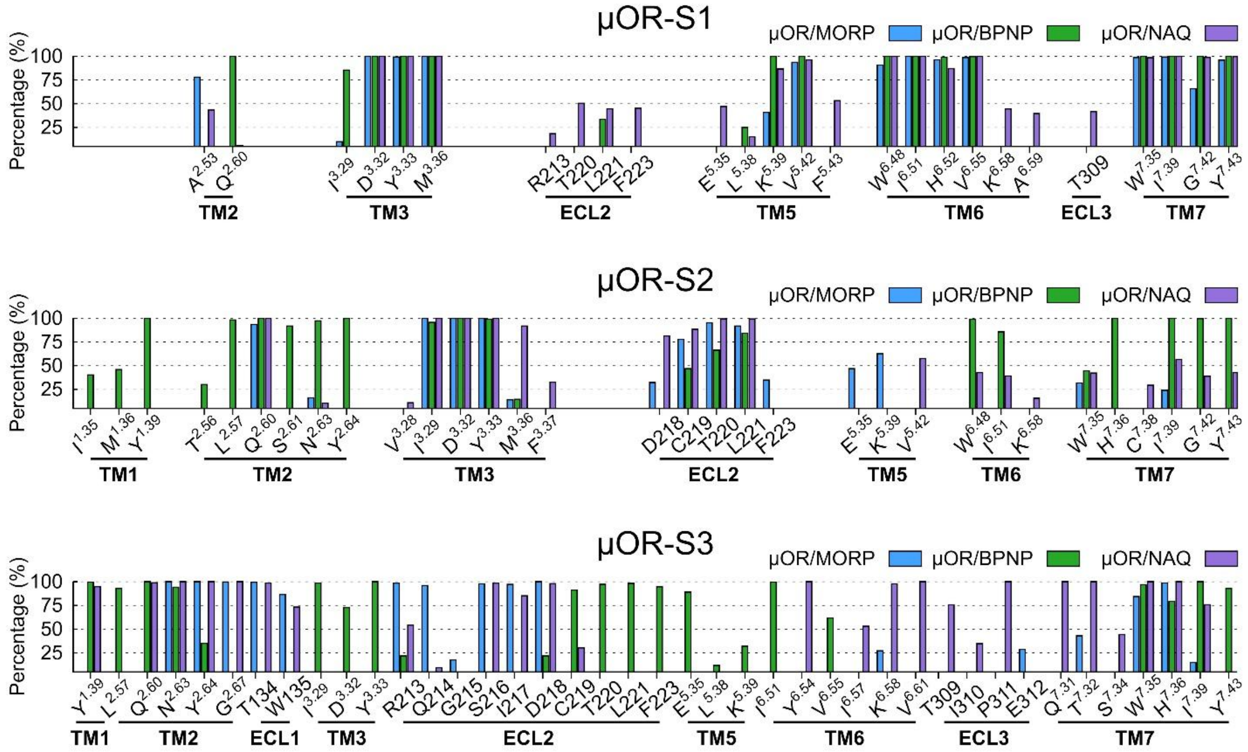

Figure S7. Percentage of contacts of ligand substates to the μOR obtained from funnel-metadynamics. Bars representing contacts in each substate (S1, S2, and S3) with morphine (MORP), buprenorphine (BPNP), and NAQ are colored blue, green, and purple, respectively. Residues are grouped according to their positions in TM and ECL regions.

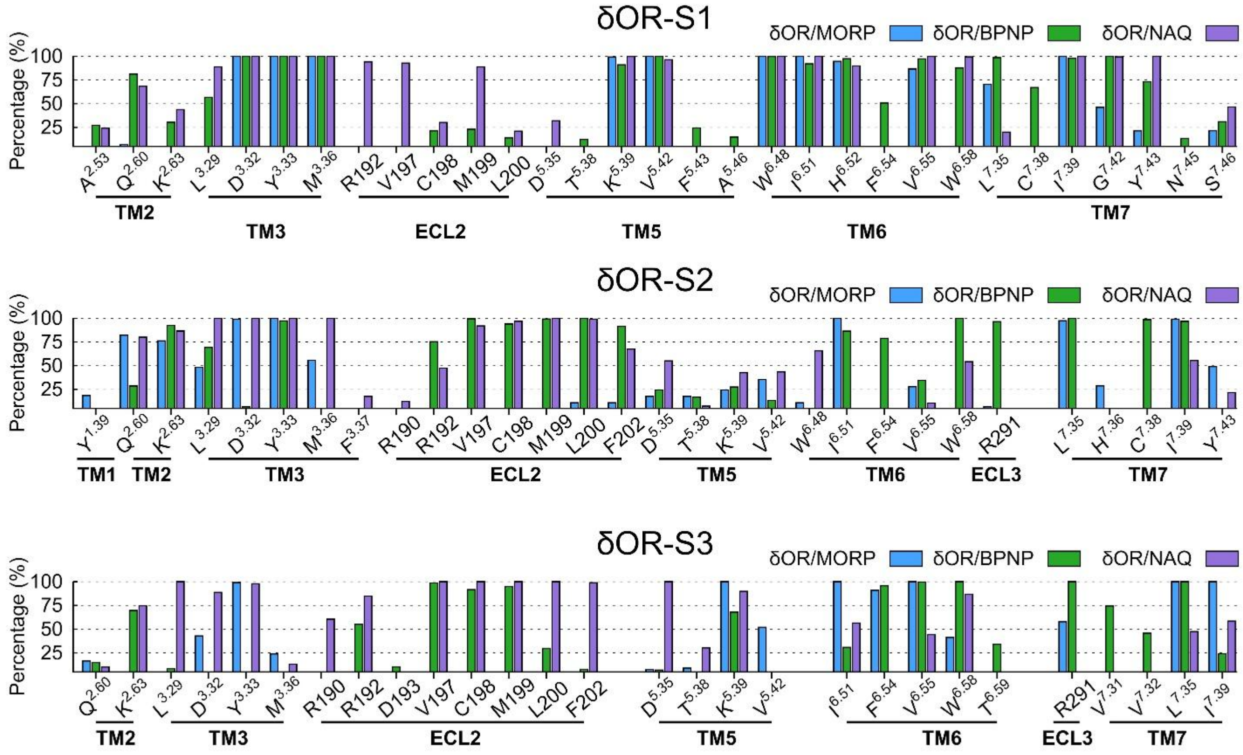

Figure S8. Percentage of contacts of ligand substates to the δOR obtained from funnel-metadynamics. Bars representing contacts in each substate (S1, S2, and S3) with morphine (MORP), buprenorphine (BPNP), and NAQ are colored blue, green, and purple, respectively. Residues are grouped according to their positions in TM and ECL regions.

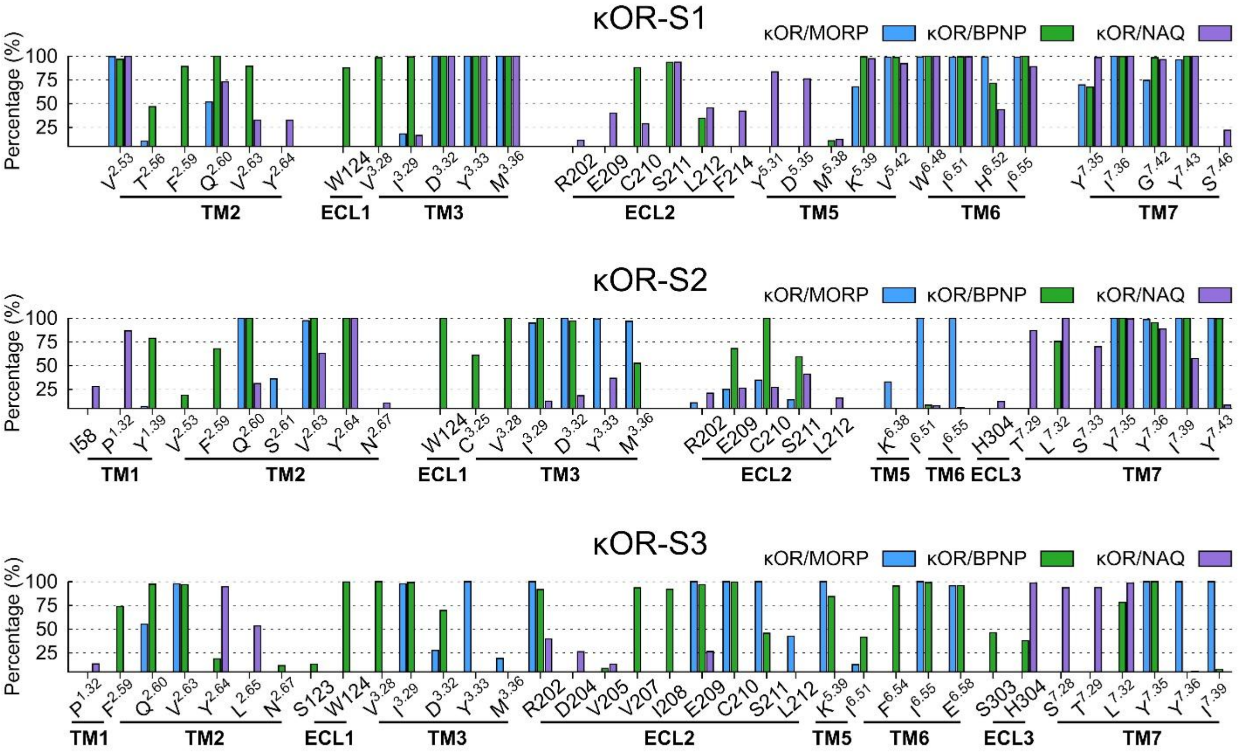

Figure S9. Percentage of contacts of ligand substates to the κOR obtained from funnel-metadynamics. Bars representing contacts in each substate (S1, S2, and S3) with morphine (MORP), buprenorphine (BPNP), and NAQ are colored blue, green, and purple, respectively. Residues are grouped according to their positions in TM and ECL regions.

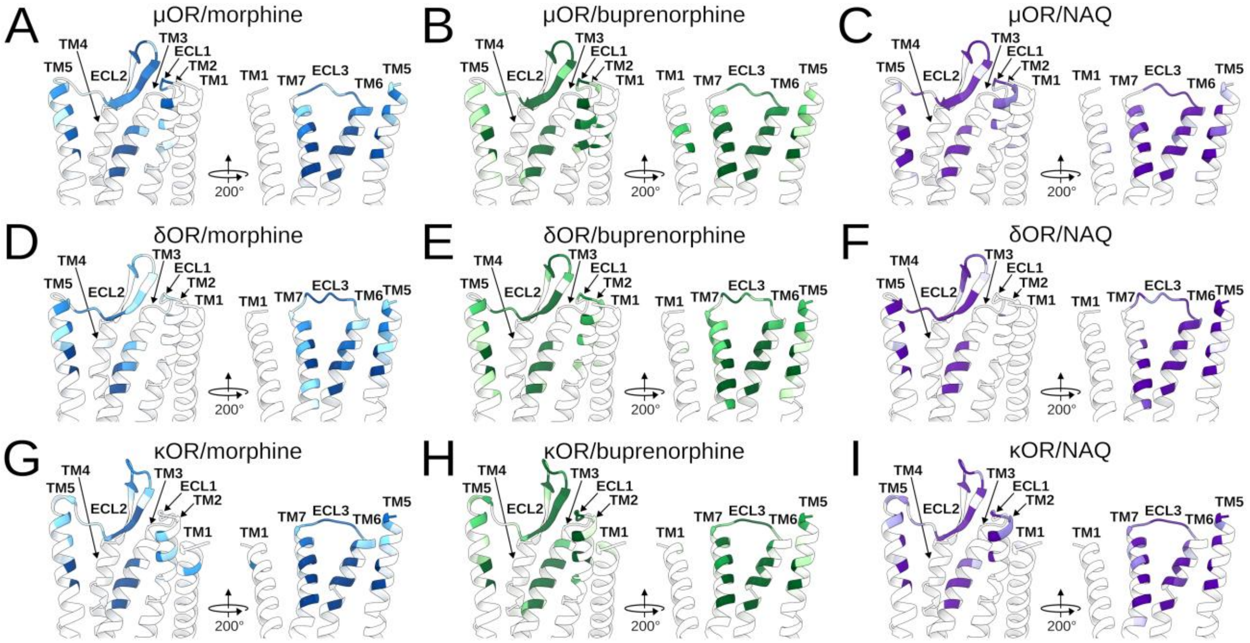

Figure S10. Identification of OR ligand-binding regions using funnel-metadynamics. Receptors are colored blue, green, and purple according to their percentages of contacts with morphine, buprenorphine, and NAQ. Stronger colors indicate higher percentages. TM or ECL portions of the receptor were omitted for clarity in visualization.

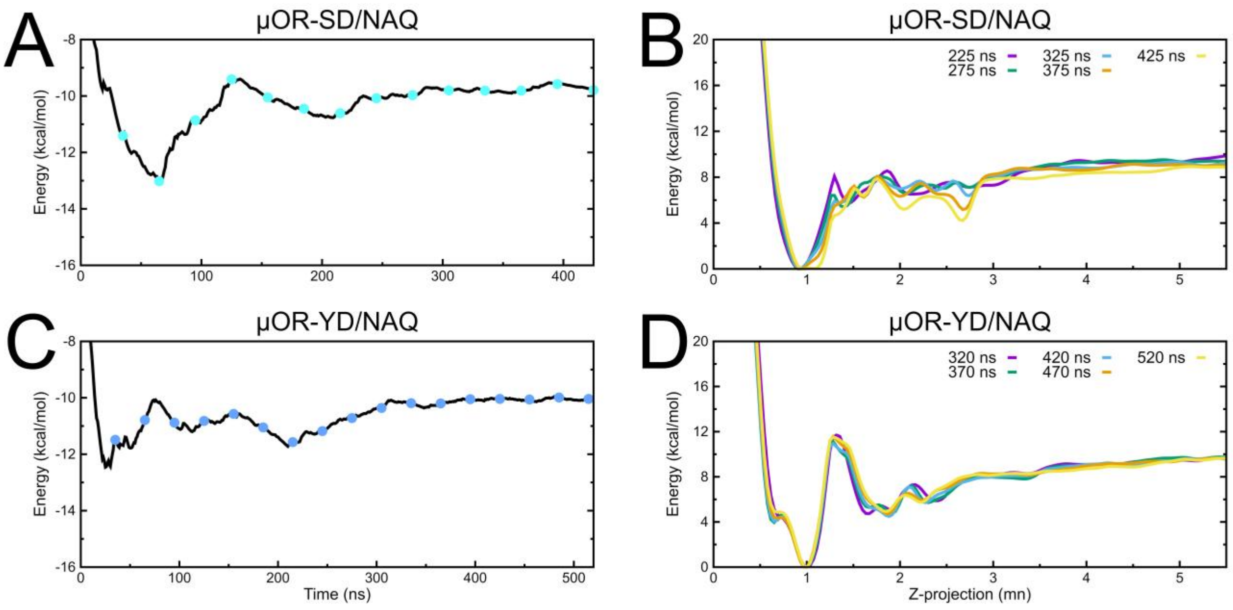

Figure S11. Free energy values of funnel-metadynamics of the μOR mutants μOR-SD ($T^{5.31}S$ and $E^{5.35}D$) and μOR-YD ($T^{5.31}Y$ and $E^{5.35}D$) bound to NAQ. Time-evolution of the computed free energy values (A and C), calculated every 1 ns, and profiles (B and D), calculated every 50 ns, show the convergence of the simulations.

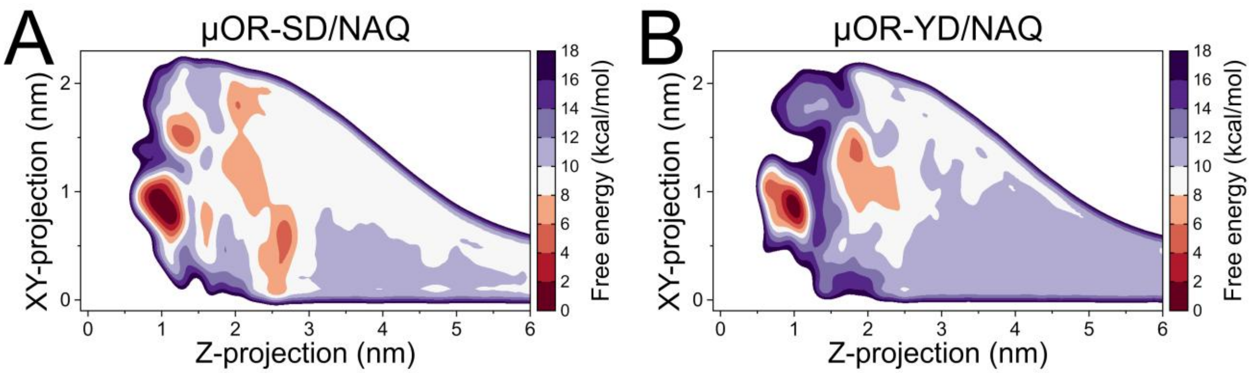

Figure S12. Free-energy landscapes for μOR mutants bound to NAQ. Mutants (A) μOR-SD ($T^{5.31}$S and $E^{5.35}$D) and (B) μOR-YD ($T^{5.31}$Y and $E^{5.35}$D) are shown in sequential palettes of purple, highlighting the presence of distinct substates. Red sequential palettes color low-energy regions.

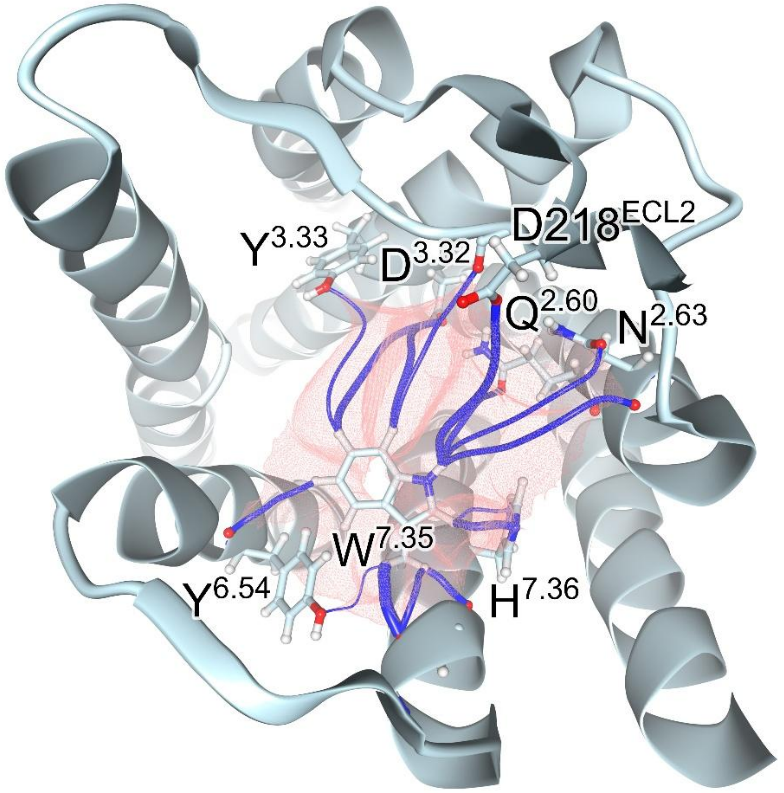

Figure S13. Electrostatic influence zone (EIZ) of the μOR residue $W^{7.35}$. The μOR is represented as light blue cartoons or sticks, the EIZ as a red mesh, and field line bundles as blue lines.

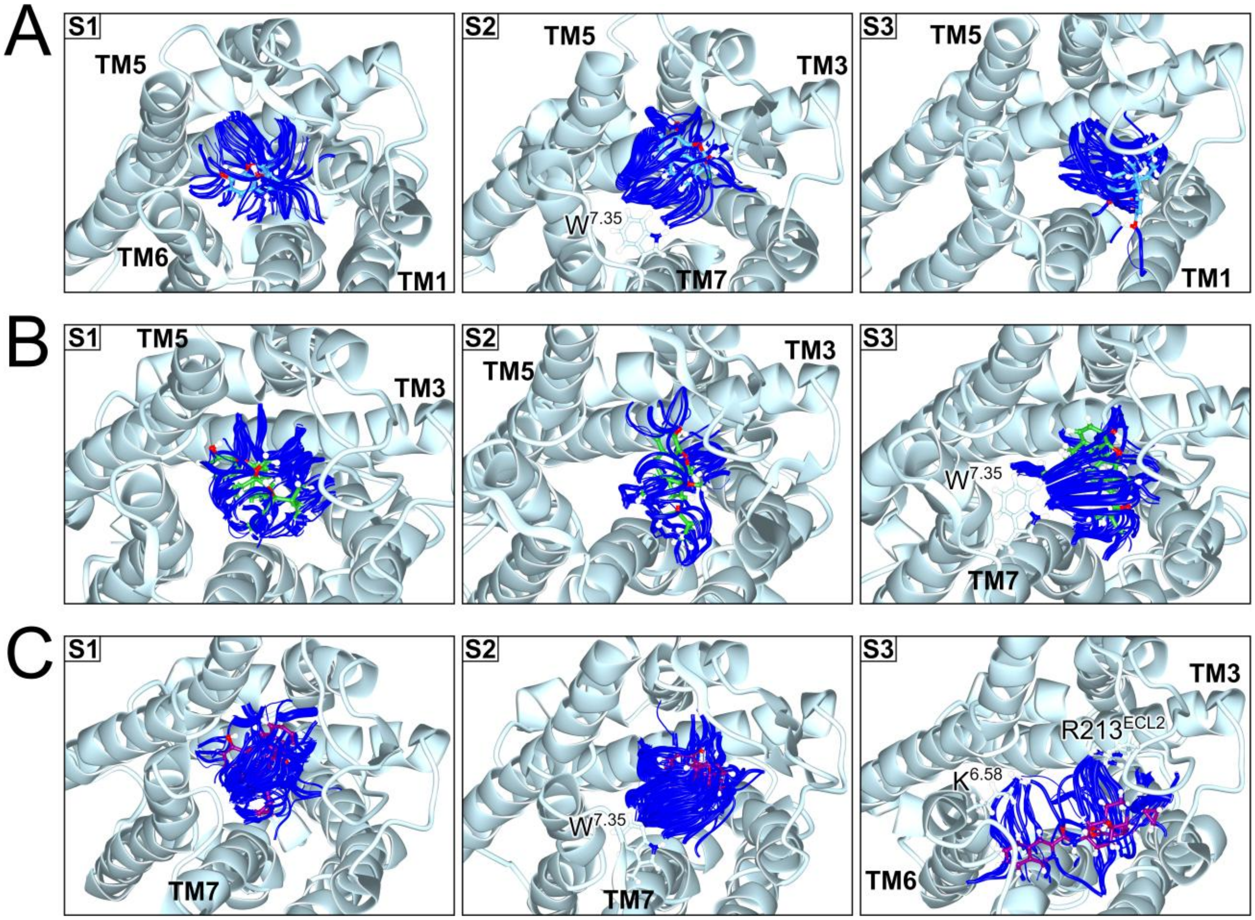

Figure S14. Electrostatic influence zone (EIZ) of ligands bound to μOR. (A) Morphine, (B) buprenorphine, and (C) NAQ substates S1, S2, and S3 are shown. The μOR is represented as light blue cartoons or sticks, while morphine, buprenorphine, and NAQ are colored blue, green, and purple sticks, respectively. EIZ bundles are shown as blue tubes. Oxygen, nitrogen, and polar hydrogen atoms are colored red, blue, and white, respectively.

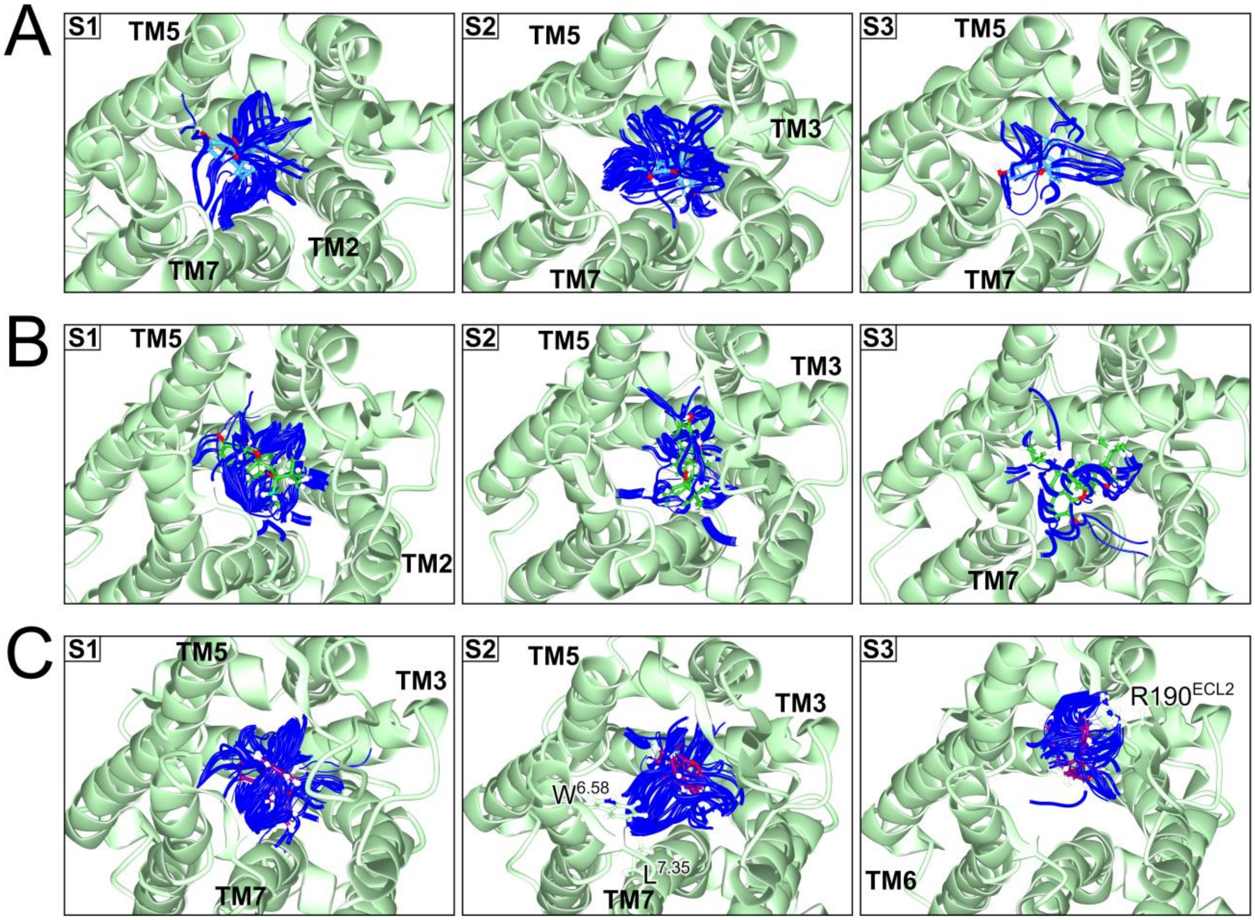

Figure S15. Electrostatic influence zone (EIZ) of ligands bound to δOR. (A) Morphine, (B) buprenorphine, and (C) NAQ substates S1, S2, and S3 are shown. The δOR is represented as light blue cartoons or sticks, while morphine, buprenorphine, and NAQ are colored blue, green, and purple sticks, respectively. EIZ bundles are shown as blue tubes. Oxygen, nitrogen, and polar hydrogen atoms are colored red, blue, and white, respectively.

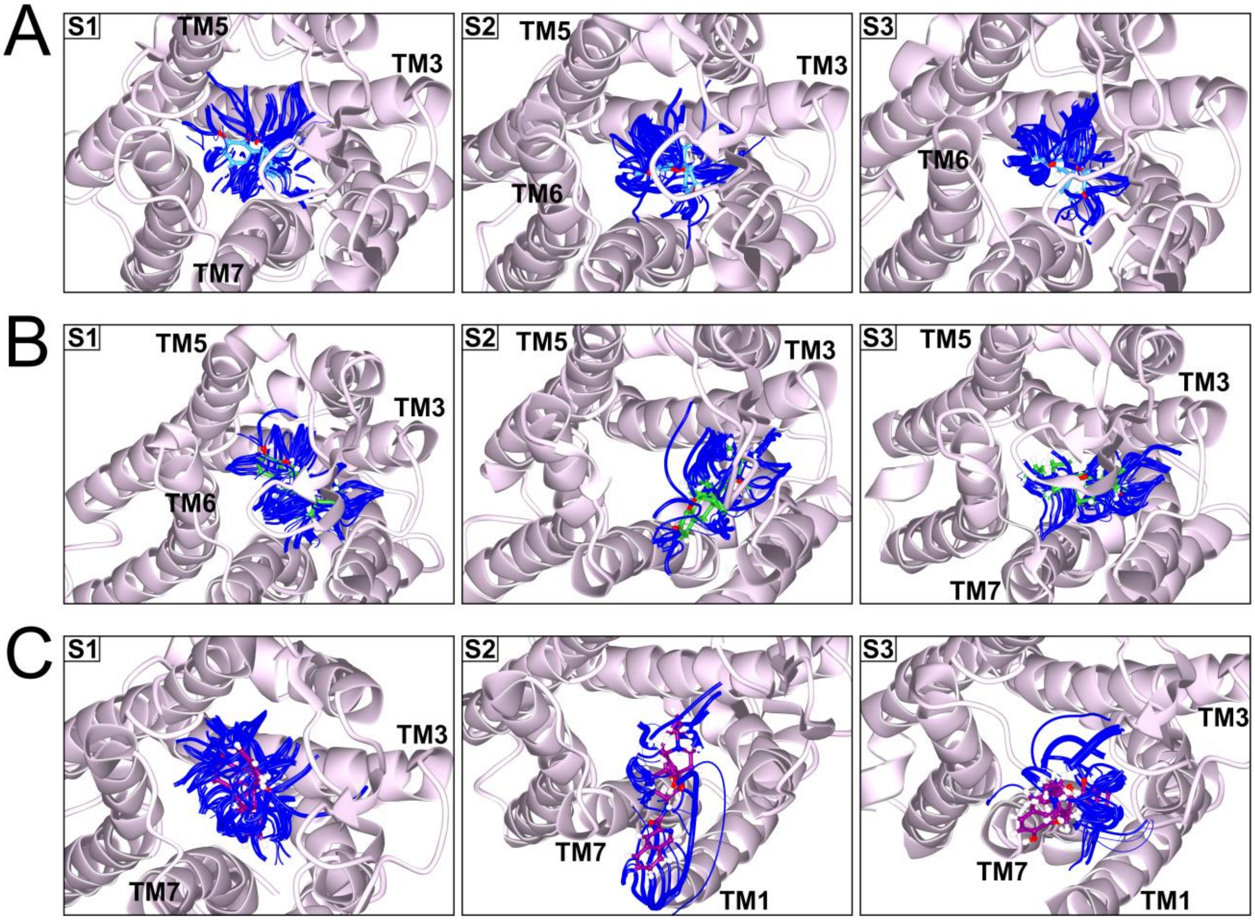

Figure S16. Electrostatic influence zone (EIZ) of ligands bound to κOR. (A) Morphine, (B) buprenorphine, and (C) NAQ substates S1, S2, and S3 are shown. The κOR is represented as light blue cartoons or sticks, while morphine, buprenorphine, and NAQ are colored blue, green, and purple sticks, respectively. EIZ bundles are shown as blue tubes. Oxygen, nitrogen, and polar hydrogen atoms are colored red, blue, and white, respectively.

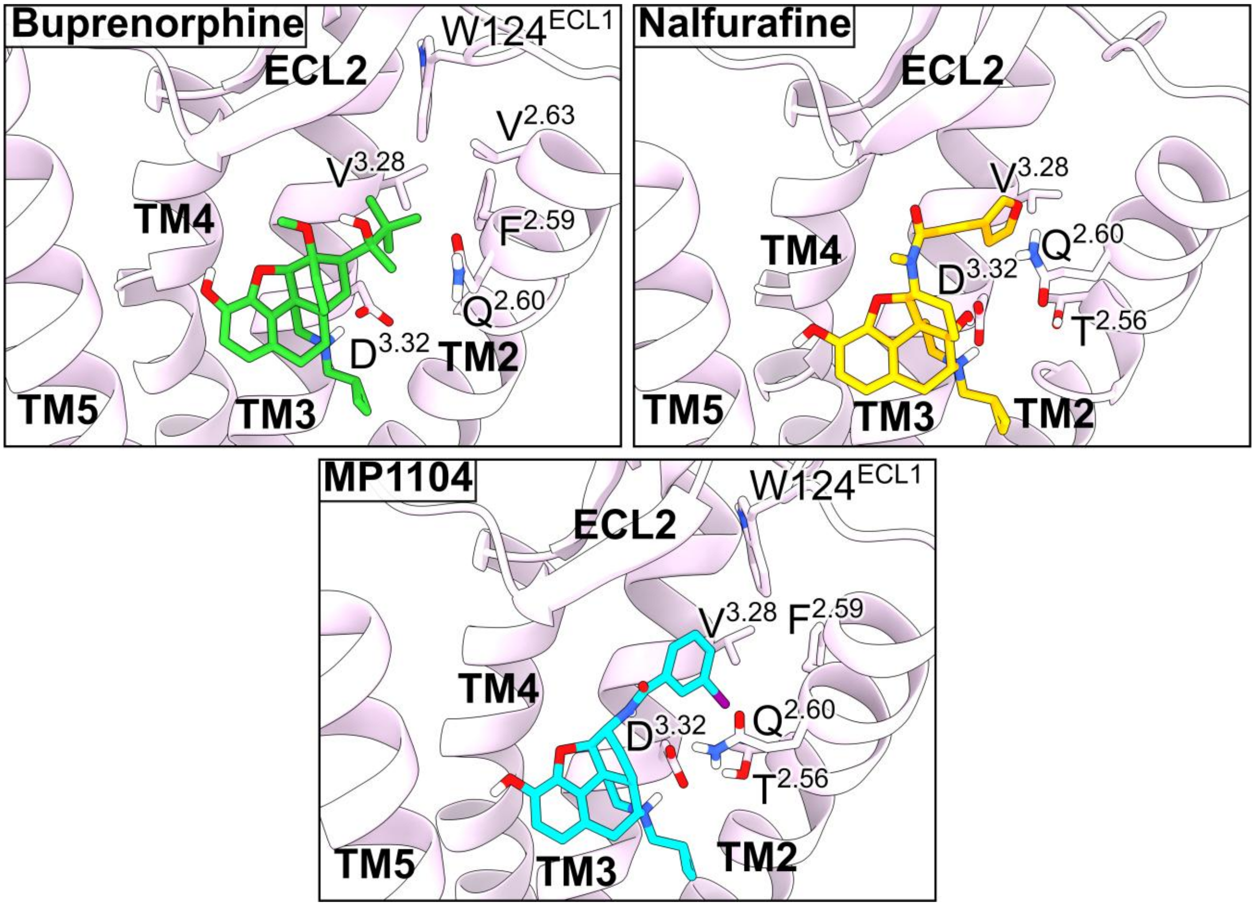

Figure S17. Structural comparison of small molecules with distinct pharmacological effects at the κOR. Interactions of buprenorphine, nalfurafine, and MP1104 with the residues of the hydrophobic subpocket formed by ECL1, TM2, and TM3 residues are shown in detail. The κOR is represented as light purple cartoons or sticks, while buprenorphine, nalfurafine, and MP1104 are colored green, yellow, and cyan sticks, respectively. Oxygen, nitrogen, iodine, and polar hydrogen atoms are colored red, blue, purple, and white, respectively. TM or ECL portions were omitted for clarity in visualization.

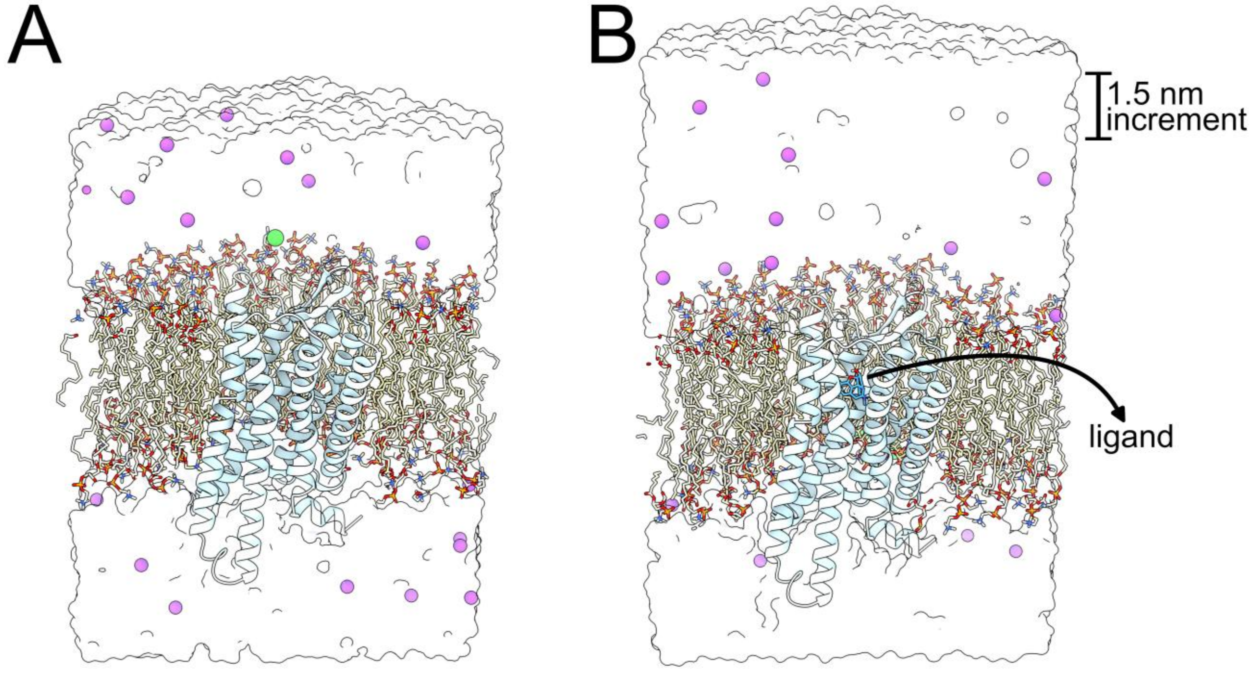

Figure S18. Representations of MD simulations boxes for (A) unbound and (B) ligand-bound systems. Systems used for metadynamics simulations are similar to unbound systems. Ligand-bound systems have a 1.5 nm increment along Z to allow ligand exploration of the unbound region. Proteins are colored light blue cartoons, while lipids and the ligand (morphine) are colored pale and blue sticks, respectively. Water molecules are represented as surfaces, with Na and Cl ions colored purple and green spheres, respectively. Oxygen, nitrogen, phosphorus, and polar hydrogen atoms are colored red, blue, orange, and white, respectively. Lipid and solvent atoms are clipped for a clearer visualization of the proteins embedded in the lipid bilayer.

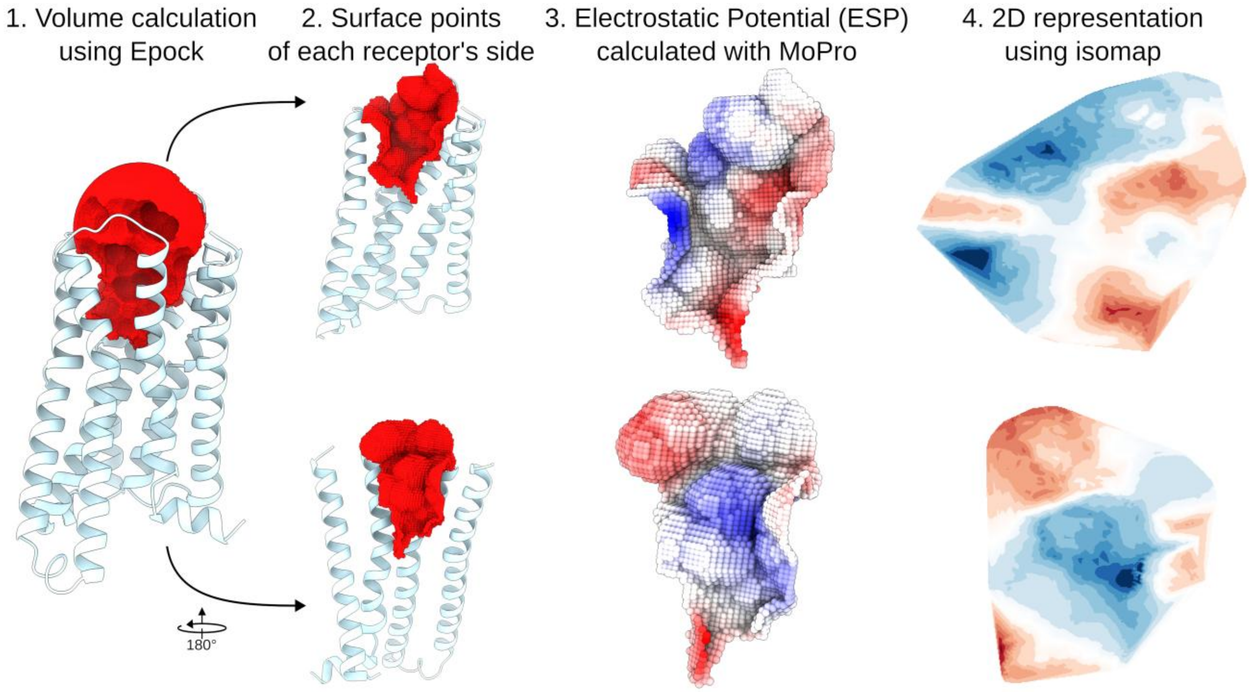

Figure S19. Strategy for representing the Electrostatic Potential (ESP) of ORs. In the example, the μOR is used to detail the steps for obtaining a 2D representation of the ESP, which consists of (1) determining the cavity shape of the μOR, then (2) defining sets of points corresponding to each side of the receptor. (3) These points received ESP values according to the electrostatic surface of the receptor atoms, which were further (4) reshaped into a 2D representation while preserving the topology of the obtained surface. The μOR is represented as light blue cartoons and the volume as red spheres. Negative, neutral, and positive ESP values are represented by red, white, and blue spheres or surfaces, respectively.

Table S1. Overview of the simulations performed. MD simulations were joined, as they present the same number of replicas and sampling time. All metadynamics simulations, including CV_{bridge} and funnel-metadynamics, were performed with multiple walkers.

OR	Starting structure	Ligand	# Atoms	Method	# Replicas	Sampling time (μs)	Total (μs)
μOR, δOR, κOR	8EF6, 8F7S, 8F7W	Unbound	65011, 65225, 67545	MD simulations	4, 4, 4	1, 1, 1	4, 4, 4
		Morphine	74534, 74643, 76759	MD simulations	1,1,1	1, 1, 1	1, 1, 1
		Buprenorphine	74704, 74714, 76986	MD simulations	1,1,1	1, 1, 1	1, 1, 1
		NAQ	74681, 74644, 76868	MD simulations	1,1,1	1, 1, 1	1, 1, 1
μOR-SD	8EF6, mutated	NAQ	74738	MD simulations	1	1	1
μOR-YD	8EF6, mutated	NAQ	74596	MD simulations	1	1	1
—	—	—		—	—	Total	23
κOR	8F7W	Unbound	67545	Metadynamics (CV_{bridge})	20	0.015	0.3
		Morphine	76759	Metadynamics (CV_{bridge})	20	0.015	0.3
		Buprenorphine	76986	Metadynamics (CV_{bridge})	20	0.015	0.3
		NAQ	76868	Metadynamics (CV_{bridge})	20	0.015	0.3
—	—	—		—	—	Total	1.2

μOR	8EF6	Morphine	74534	Funnel-Metadynamics	20	0.025	0.5
		Buprenorphine	74704	Funnel-Metadynamics	20	0.030	0.6
		NAQ	74681	Funnel-Metadynamics	20	0.040	0.8
		μOR-SD/NAQ	74738	Funnel-Metadynamics	20	0.0212	0.425
		μOR-YD/NAQ	74596	Funnel-Metadynamics	20	0.026	0.520
δOR	8F7S	Morphine	74643	Funnel-Metadynamics	20	0.020	0.4
		Buprenorphine	74714	Funnel-Metadynamics	20	0.030	0.6
		NAQ	74644	Funnel-Metadynamics	20	0.045	0.9
κOR	8F7W	Morphine	76759	Funnel-Metadynamics	20	0.025	0.5
		Buprenorphine	76986	Funnel-Metadynamics	20	0.040	0.8
		NAQ	76868	Funnel-Metadynamics	20	0.030	0.6
—	—	—		—	—	Total	6.645
